# Questionnaires in otology: a systematic mapping review

**DOI:** 10.1186/s13643-021-01659-9

**Published:** 2021-04-20

**Authors:** Koen Viergever, Jeroen T. Kraak, Els. M. Bruinewoud, Johannes C. F. Ket, Sophia E. Kramer, Paul Merkus

**Affiliations:** 1 Amsterdam UMC, Vrije Universiteit Amsterdam, Otolaryngology – Head & Neck Surgery, Ear & Hearing, Amsterdam Public Health research institute, De Boelelaan, 1117 Amsterdam, Netherlands; 2 Medical Library, Vrije Universiteit Amsterdam, De Boelelaan, 1117 Amsterdam, Netherlands

**Keywords:** Questionnaires, Patient-reported outcome measures (PROMs), Otolaryngology, Ear diseases

## Abstract

**Background:**

Patient-reported outcome measures (PROMs) are valuable tools in assessing the quality of health care from a patient perspective and are increasingly used by otologists. However, selecting the right questionnaire has proven to be a difficult and time-consuming task. To facilitate this process, we will provide a comprehensive overview of existing questionnaires.

**Methods:**

A systematic literature search has been conducted on August 26, 2019, using the EMBASE and PubMed medical databases. 13,345 unique records were extracted. Questionnaires addressing any otologic complaint (tinnitus, hearing loss, earache, otorrhoea, and ear-related pressure sensation, vertigo, itch, or dysgeusia) were identified. All questionnaires were evaluated for eligibility by two independent researchers. Inclusion criteria were adult population, closed-ended questions, English language of the questionnaire, and the availability of the original article describing the development of the instrument or a validation paper describing the validation process written in English.

**Objective:**

Create a comprehensive overview of all validated closed-ended otology questionnaires for adults and demonstrate their basic characteristics.

**Main outcome measure:**

The number of questionnaires in English literature for the adult population, subdivided per symptom and target population.

**Results:**

A total of 155 unique questionnaires were selected: 33 tinnitus questionnaires, 23 vertigo questionnaires, 84 hearing loss questionnaires, and 15 multiple complaint questionnaires. A protocol for further questionnaire comparison is presented.

**Discussion:**

Two separate sequential searches were needed to identify unique questionnaires and to identify their development/validation paper. Although many ear diseases create multiple symptoms, the majority of the questionnaires were symptom specific.

**Conclusion:**

Many questionnaires concerning ear-related symptoms exist and predominantly concern hearing loss, vertigo, or tinnitus. Only a few questionnaires cover the multiple complaints that ear diseases can create. The presented overview is the most comprehensive overview of otology questionnaires in literature to date. It will serve as a basis for questionnaire selection by professionals and could serve as a protocol for questionnaire selection in other fields.

**Systematic review registration:**

PROSPERO CRD42017058155

**Supplementary Information:**

The online version contains supplementary material available at 10.1186/s13643-021-01659-9.

## Background

A patient-reported outcome measure (PROM) gathers information obtained directly from patients without any interference from others. The terms PROM and questionnaire are often being used interchangeably although not every questionnaire is a PROM [1]. Questionnaires have great value in the collection of subjective outcome measures (e.g., perceived disability) as it is a fast and cost-effective method compared to the only alternative, a patient interview. The need for subjective outcome measures has been demonstrated in studies showing that outcomes of objective measurements do not necessarily correlate with the patients’ subjective experience (e.g., audiometric results vs. perceived hearing disability) [2, 3]. Furthermore, the burden of disease is not only the direct result of symptoms, as it can be heavily affected by accompanying cognitive and emotional factors (e.g., the fear of a vertigo attack in between two episodes), as well as environmental factors. This may cause two persons with identical disease activity to suffer from different degrees of disability. Besides quantifying subjective symptoms, questionnaires give insight into what aspects of life a patient has complaints about, or within which disability is experienced (e.g., social interactions, work). This allows caregivers to customize therapy according to the specific needs of the patients.

The shift towards patient-centered care in modern health care is followed by an increasing demand for validated questionnaires. The need for otology questionnaires is further demonstrated by otological conditions, like otitis media and hearing loss, being world-wide among the most common short-term and chronic diseases, respectively. Age-related hearing loss is measured to have a greater impact on global health than asthma or lung carcinoma, with future incidence expecting to rise [4].

Many new questionnaires have been developed in the last decades resulting in a substantial amount of literature. A thorough review of this literature is a time-consuming process. Systematic reviews on questionnaires used in tinnitus, vertigo, or hearing loss do exist, although recently performed comprehensive studies are rare. Furthermore, many of these studies provide an overview of existing questionnaires without any additional information about the questionnaire itself. This is demonstrated by the available literature in the field of Audiology. Both Granberg et al. [5] and Akeroyd et al. [6] have conducted a (systematic) search for PROMs in hearing loss and present a list of often used questionnaire titles. However, no information about the questionnaire itself is presented.

We believe the questionnaire is a vital tool for the assessment of complaints in ENT patients. However, questionnaire selection is often guided by prior experiences or by copying from the work of peers. The most suitable questionnaire is not always selected [7]. A comprehensive overview of all published otology questionnaires demonstrating basic questionnaire and study characteristics will be a valuable addition to the current literature. It will facilitate the selection of questionnaires by caregivers. In the presented article, we will describe the systematic search of all closed-ended questionnaires, validated in adults, regarding at least one of the eight most prominent ear complaints [8] like tinnitus, hearing loss, earache, otorrhoea, and ear-related vertigo, pressure sensation, prurigo, or dysgeusia. A distinct overview of questionnaires will be presented with their general characteristics, divided into several subgroups. More in-depth assessment of the reliability/validity of every questionnaire in each group will follow in future studies.

### Objectives

1. To identify all validated closed-ended questionnaires covering one or multiple ear complaints (i.e., tinnitus, hearing loss, otalgia, otorrhoea, and ear-related pressure sensation, vertigo, prurigo, or dysgeusia) for adults, in the literature published in the English language.
2. Create a comprehensive overview of identified questionnaires and their basic characteristics.

## Methods/design

### Protocol and registration

The methods for this systematic mapping review have been developed according to the recommendations from the Preferred Reporting Items for Systematic Reviews and Meta-Analyses (PRISMA) statement [9]. A 27-item PRISMA checklist is available as an additional file to this protocol (Additional file 1). Our protocol has been registered in the International Prospective Register of Systematic Reviews (PROSPERO): CRD42017058155.

### Search strategy

**Fig. 1:**
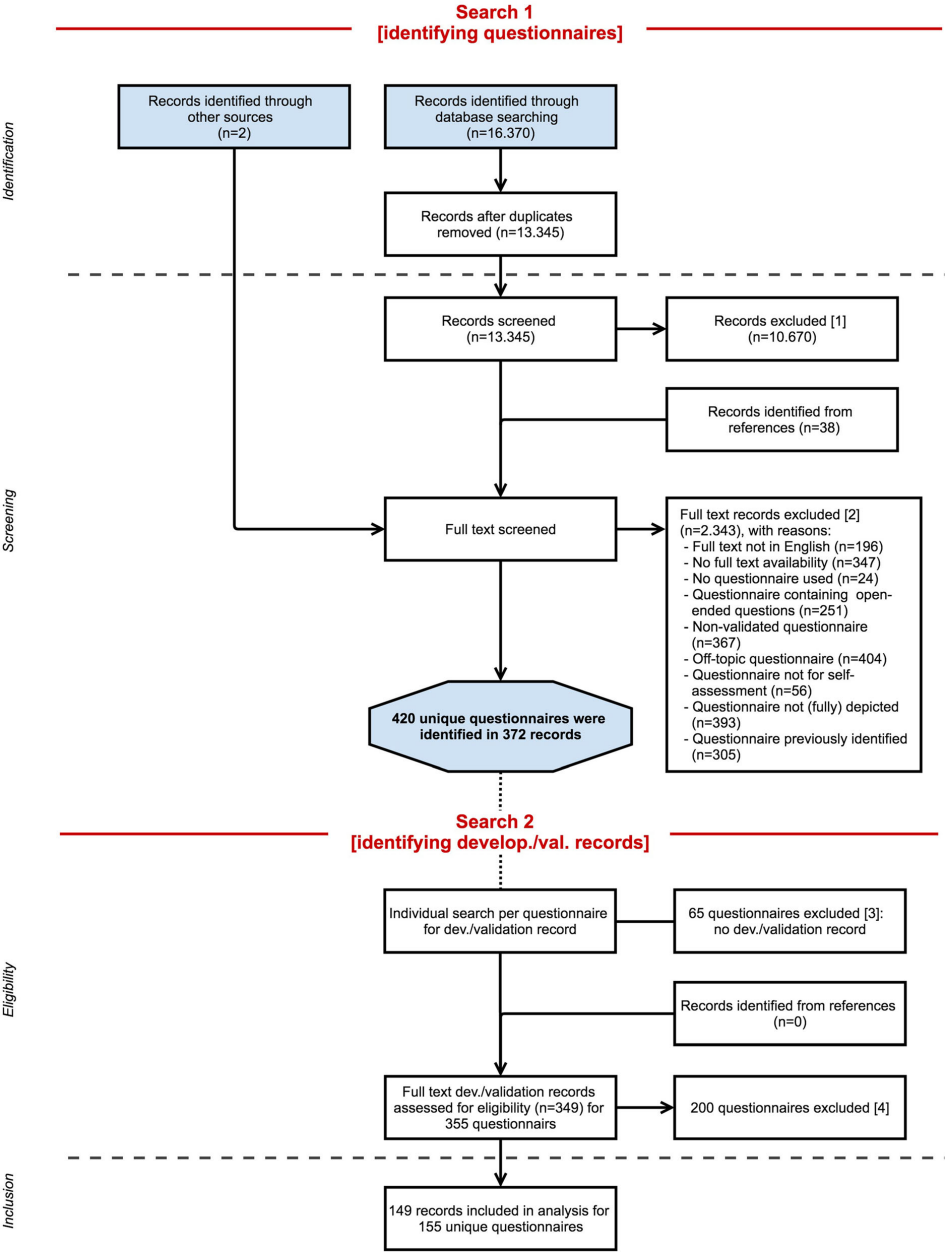
PRISMA flowchart illustrating the study selection process. Search 1 encompasses the identification of questionnaires through screening of the records originating from the database searches and by expert recommendation. Search 2 encompasses the eligibility assessment of the potentially relevant questionnaires by identifying and assessing their development and/or validation paper. This resulted in the inclusion of validated questionnaires. Records could be excluded at every step of the process, after screening of the title and abstract of the database record [1], full-text assessment of the database record [2], failure to identify a development or validation paper [3], and during eligibility assessment of development or validation paper [4]

The search strategy is illustrated using a flow diagram (Fig. 1). Our search goal was to identify all otology questionnaires and subsequently to identify any original article concerning the development (“development paper”) and/or an article describing the evaluation of the measurement properties (“validation paper”) for every unique questionnaire. Therefore, the search consisted of two separate searches. The goal of the first search (Search 1) was questionnaire identification. Study input for Search 1 originated from 2 sources. The first source is “records identified through database searching.” A systematic literature search was performed in two bibliographic database sources, PubMed and Embase.com. The queries included indexed terms and free-text words and synonyms, for example “questionnaire,” “ear ache,” “hearing loss,” or “tinnitus.” The queries excluded “children” and “animal studies” (the complete search queries can be seen in Additional file 2).

The second source “records identified through other sources” was added. This could be any other source, e.g., journals not included in PubMed or Embase.com. This also included questionnaires suggested by experts in the field of otology and audiology at Amsterdam University Medical Centre, location Vrije Universiteit Medical Center whom were asked to submit additional questionnaires, when this questionnaire was not found via the systematic search. Search 1 produced a set of records (i.e., study articles) that were screened in order to identify every otology questionnaire.

At the end of this stage, the screening of the 2 sources created a list of potentially eligible otology questionnaire names. The identification of corresponding development/validation articles was not part of this stage of the search.

The goal of the second search (Search 2) was to identify a development and/or validation article for all the questionnaires identified in Search 1. Thus, for every questionnaire identified in Search 1, a separate search (Search 2) was performed in the PubMed and/or Embase.com databases. Search 2 started with examining the references corresponding to the questionnaires identified in our primary search. The study title, questionnaire name, questionnaire abbreviations or acronyms, or author name(s) were entered between double quotation marks as search term. This often resulted in iterative reference searching. At the end of Search 2, a development and/or validation article was identified for every included questionnaire.

The first systematic search (Search 1) was performed in PubMed and Embase.com on January 1, 2016, by KV and JCFK. Search 2 was performed during the following months of eligibility assessment. The database search was repeated on August 26, 2019. We did not apply any restriction regarding the original publication date, language of publication, or study type of the papers in both searches. In both searches, additional study input could originate from the reference lists of assessed articles, “records identified from references” (Fig. 1). However, reference list checking was not routinely performed on all records. Exclusion of records could take place at every step in the study selection process. Search results were entered into reference management software (EndNote version X9, Clarivate Analytics, Philadelphia, PA).

### Eligible studies

All PROMs regarding tinnitus, hearing loss, otalgia, otorrhoea, and/or ear-related pressure sensation, vertigo, prurigo, or dysgeusia, or all synonyms, in adults, presented in a full-text paper that concerns the development (“development paper”) and/or evaluation of the measurement properties (“ validation paper”) were eligible for inclusion. These symptoms were defined in earlier research [8]. Adult age is defined as 18 years or older. Questionnaires that were validated for an adult population including a small proportion of teenagers were also included, as we do not believe this immediately invalidates the questionnaire. All items in the questionnaire had to be closed-ended. Proxy-reported questionnaires or questionnaires assessing complaints in proxies (e.g., spouses) caused by patients’ disease were not considered eligible. Although generic questionnaires (e.g., generic QoL questionnaires) are applicable to the otology population, they were not considered eligible, as they do not specifically address the issues important for otology patients [10]. However, questionnaires addressing the effect of otologic symptoms/diseases on a patient’s quality of life, disease-specific health-related quality of life (HR-QoL) questionnaires, were eligible for inclusion. Questionnaire language had to be English. Otherwise, questionnaires were eligible for inclusion provided that they have been validated, with full-text availability of the development and/or validation paper in the English language. All exclusion criteria were applied to Search 1, together with the inclusion criteria “questionnaire addressing otology complaint.” All inclusion and exclusion criteria (Table 1) were applied to Search 2.

**Table 1:** Inclusion and exclusion criteria used for the assessment of questionnaire eligibility as used in Search 2

Inclusion criteria	Exclusion criteria
Exclusively closed-ended questions	Presence of open-ended questions
Adult population (≥ 18 yrs)	Pediatric population (< 18 yrs)
Copy of questionnaire available	No copy of the questionnaire available
Availability of a development or validation paper in English	No availability of a development or validation paper in English
Questionnaire addressing otology complaints	Proxy-reported questionnaires

### Article selection

All search records from Search 1 were screened by two independent reviewers (KV and EB) for eligibility. This process was aimed at identifying questionnaires through screening of the title and abstract. Full text was examined in case of doubt over eligibility. Questionnaire identification was followed by identification of the corresponding development paper and/or validation paper (Search 2). Eligibility was assessed according to the criteria depicted in Table 1. This was performed by one reviewer. Full-text copies of potentially relevant original articles and/or validation papers were purchased. Any disagreement between the reviewers over the eligibility of particular articles was resolved through discussion between the reviewers. When needed, our expert team discussed selection difficulties.

### Data extraction

Data extraction was conducted by one reviewer using a standardized extraction form and was checked by a second reviewer. This standardized extraction form is based on the patient-reported outcome (PRO)-specific checklist derived from the European Regulatory Issues on Quality of Life Assessment Working Group (ERIQA) [11].

Data extraction pertained to the secondary outcome measures: measurement properties and questionnaire characteristics of the identified questionnaires. The full extraction form is available as Additional file 3. Outcomes used in this article were first author, year of publication, country of development, institution of development, number of items, means of item generation, demographics of the study population, and available validated translations. An overview of the other questionnaire characteristics that were extracted from the development and/or validation papers is shown in Additional file 3 and is related to the measurement properties and psychometric characteristics of these questionnaires (e.g., questionnaire (sub)domains and test-retest reliability). The psychometric data will be separately assessed per symptom category in future work.

## Results

The majority of identified questionnaires were found through our database search. The PubMed and Embase.com searches resulted in a total of 16,370 records. After removal of duplicates, 13,345 unique records remained. In 10,670 cases, screening of the title and abstract was sufficient for record exclusion. However, the screening phase was primarily aimed at identifying potentially relevant questionnaires. Two records were added through expert recommendation.

Full-text examination was performed if the use of a questionnaire could not be excluded on the basis of title and abstract. A total of 2715 articles were fully assessed. At this stage, exclusion of records (Fig. 1 “records excluded [1] and[ 2]”) was primarily because both the abstract and full-text publication of the article were not accessible, full-text language was not English, the study was off-topic (e.g., not in the field of otology, pediatric population), or because of the absence of a questionnaire.

Full-text examination resulted in the exclusion of many “anonymous” questionnaires. These concerned questionnaires without a name, in the absence of a graphic representation, and without any information on the development and/or validation or reference to a corresponding article. Only records without a potentially eligible questionnaire were excluded. Two thousand three hundred forty-three records were excluded based on full-text examination out of the 2715 records. In total, 420 questionnaires were identified as potentially eligible.

In the following phase, all 420 questionnaires were assessed for eligibility. From this point, data on all questionnaires were saved in a separate digital database including the reasons for questionnaire exclusion (Fig. 1: “records excluded [3] and [4]”). Eligibility was assessed by examination of the development and/or validation article. A total of 265 questionnaires were excluded for various reasons (Table 2), and the most common reason being that full text of the development and/or validation article could not be retrieved (*N* = 65). Excluded questionnaires were categorized according to symptoms. The remaining 155 questionnaires were included. One hundred forty-nine studies were included in our analyses, because several studies presented more than one questionnaire. The questionnaires were categorized based on the primary symptom of interest. This resulted in the following classification: 33 tinnitus questionnaires, 23 vertigo questionnaires, 84 hearing loss questionnaires, and 15 multiple complaint questionnaires (i.e., ≥3 symptoms of interest). Within these symptom categories, a subdivision was made based on the target population of the different questionnaires (Table 3). The majority of identified hearing loss questionnaires were designed for patients with sensorineural hearing loss using hearing aids or cochlear implants. Questionnaires for patients with autophony, hyperacusis, and patulous Eustachian tube completed this category. Thirty-three questionnaires have been designed for tinnitus sufferers. Unlike hearing loss questionnaires, tinnitus questionnaires do not pertain to specific patient groups, e.g., vestibular schwannoma or Meniere’s disease patients. Questionnaires specific for vestibular schwannoma or Meniere’s disease patients do exist. However, these questionnaires, together with symptom-specific questionnaires on otitis media, Eustachian tube dysfunction, and superior canal dehiscence, were categorized as multiple complaint questionnaires. Other multiple complaint questionnaires focused on dizziness, or cochlear implantees, or were designed as generic otology questionnaires. Most vertigo questionnaires did not relate to a specific condition. However, disease-specific questionnaires for benign paroxysmal positional vertigo, visual vertigo, persistent postural-positional vertigo, motion sickness, and simulator sickness were also identified. An overview of all included questionnaires is presented together with the first author name, year of publication, institution and country of development, a description of the construct assessed by the questionnaire, demographics of the study population of the development/validation study, methods of item generation, and the existing validated translations (Additional files 4, 5, 6 and 7). However, the following conclusions can be drawn from the data as reported in the Additional files 4, 5, 6 and 7. First, in every symptom category, more questionnaires have been developed in the last 20 years than prior to this period: tinnitus (23 out of 33 were developed between 2000 and 2020), vertigo (13 out of 23), hearing loss (50 out of 84), and multiple complaints (13 out of 14). Long questionnaires (<60 items) are no longer conventional. In general, newly developed questionnaires are shorter (<40 items) and many existing questionnaires have been shortened. Furthermore, existing questionnaires are often used in the development of new questionnaires. Some questionnaires are composed out of items from various existing questionnaires, and sometimes a new questionnaire is generated by replacing a single word (i.e., the construct of interest) in every item of an existing questionnaire.

**Table 2:** Numbers of studies excluded per diagnostic group and reasons for exclusion. Each column represents a different symptom category

	Tinnitus	Vertigo	Hearing loss	Multiple complaint	Dysgeusia	Itch
No development/validation paper	13	15	34	3	-	-
Open-ended question(s)	7	2	25	-	-	-
Q not self-administered	1	3	3	1	-	-
No description of development/validation in OA	4	6	19	3	3	1
No definitive Q presented	1	-	-	-	-	-
Off-topic	1	11	15	2	-	-
No English translation	3	2	3	-	-	-
No Q availability	1	6	12	1	-	-
Pediatric Q	-	-	8	5	-	-
Not a Q	1	3	8	-	-	-
1-item Q	3	3	1	-	-	-
Double Q	9	10	11	-	-	-
Semi-structured Q	-	-	2	-	-	-
**Total (*n*** **= 265)**	**44**	**61**	**141**	**15**	**3**	**1**

*Q* questionnaire, *OA* original article. See also Fig. 1, Search 2, exclusions [3] and [4]

**Table 3:** The selected 155 questionnaires subdivided per symptom category

Tinnitus (***n***=33)	Vertigo (***n***=23)	Hearing loss (***n***=84)	Multiple complaint (***n***=15)
Non-specified tinnitus (*n*=33)	Non-specified vertigo (*n*=14)	Hyperacusis (*n*=4)	Meniere’s disease (*n*=3)
	Simulator sickness (*n*=2)	Hearing impaired (*n*=32)	Otitis media (*n*=3)
	Motion sickness (*n*=2)	Hearing aid users (*n*=16)	Vestibular schwannoma (*n*=1)
	Benign paroxismal positional vertigo (*n*=3)	Cochlear implant users (*n*=11)	Eustachian tube dysfunction (*n*=1)
	Persistent postural-positional vertigo (*n*=1)	Geriatric population/older adults (*n*=2)	General otology population (*n*=2)
	Visual vertigo (*n*=1)	Autophony (*n*=2)	Superior canal dehiscence (*n*=1)
		Hearing aid candidates (*n*=4)	Dizziness sufferers (*n*=1)
		General population (*n*=12)	General population (*n*=2)
		Otology patient, not further defined (*n*=1)	Cochlear implant users (*n*=1)
		Patulous Eustachian tube (*n*=1)	

## Discussion

Questionnaires are essential for the assessment of subjective complaints and disability in the otology population. To date, no comprehensive overview of all, in English available, structured otology questionnaires for adults exist. As presented in this article, 155 unique validated questionnaires were identified, classified into four categories. These categories are tinnitus questionnaires, vertigo questionnaires, hearing loss questionnaires, and multiple complaint questionnaires (i.e., ≥3 symptoms of interest), containing 33, 23, 84, and 15 unique questionnaires, respectively. Questionnaires were categorized according to symptom and target population (Table 3) demonstrating a great variety between and within symptom categories. There is no subdivision within the tinnitus category. Thus, all 33 questionnaires are eligible for every tinnitus patient. The other 3 symptom categories do contain subcategories. In contrast to “tinnitus,” the “hearing loss” category has 10 subgroups with varying numbers of questionnaires. Table 3 gives a clear overview of the number of questionnaires available and shows how many questionnaires can be subdivided into different (sub)categories. We have not identified a similar overview in the literature on questionnaires in otology. Supported with the questionnaire characteristics presented in Additional files 4, 5, 6, and 7, clinicians are facilitated in the selection of an appropriate questionnaire, being able to select a questionnaire based on its construct, number of items, or the availability of a validated translation.

In order to determine the accuracy of our search, it is necessary to compare our work to various other (systematic) reviews that all pertain to a specific category within the topic of otology questionnaires.

### Hearing loss

First, a comprehensive review by Akeroyd et al. [6] of all adult hearing loss questionnaires reported a total of 139 questionnaires. These results were exceeded in our search (i.e., 228 hearing questionnaires prior to eligibility assessment). However, the absence of information on the methods or the identified questionnaires did not allow further comparison.

Granberg et al. [5] performed a systematic review on outcome measures used in adults with hearing loss: 34 condition-specific questionnaires were presented. The majority of these questionnaires were identified in our search: 23 out of 34. A possible explanation for the difference in identified questionnaires could be the difference in databases that were consulted. Granberg et al. consulted 7 databases (i.e., PsycInfo, CINAHL, AMED, ERIC, Sociological Abstracts, PsycArticles, and CENTRAL) in addition to PubMed and Embase.com.

The scoping review by Barker et al. [12], on outcome measurements used in randomized clinical trials in adult auditory rehabilitative research, identified 13 hearing questionnaires. All except the 2 questionnaires focused on communication (the Primary Communication Inventory [13] and the Communication Scale for Older Adults [14]) were identified in our results. Most likely as our research and search focused on ‘hearing’ as a complaint in daily ENT practice, ‘communication’ did not match with synonyms or had any ‘hearing’ overlap.

No description of questionnaire (development) characteristics was given by Barker et al., Granberg et al., or Akeroyd et al. Therefore, these studies appear less useful for questionnaire selection purposes compared to this study.

### Multiple complaints

The definition “multiple complaint questionnaire” (i.e., ≥3 ear-related symptoms) is not conventional in questionnaire literature as we have defined this for the purpose of this study. This is because in many ear diseases, prior or post-treatment, many patients complain about 3 or more symptoms. This is also seen in the recent development and use of more generic otology questionnaires when used in a population with all kinds of diseases of the ear (OQUA [8], COQOL [15]). Questionnaires that inquire information about “multiple complaints” are usually designed as disease-specific questionnaires. This is supported by the outcome of our study. Out of the multiple complaints group, 9 were disease-specific questionnaires compared to 4 general otology questionnaires. Our study design did not limit our search results to a particular disease. Therefore, it enabled us to identify questionnaires that do not pertain to a specific disease or patient group (e.g., tinnitus patients), hence the identification of the 4 general otology questionnaires. Two of these questionnaires were designed as screening instruments, assessing the need for referral to an otologist for individuals in the general population. The other 2 questionnaires are the Cambridge Otology Quality of Life Questionnaire (COQOL) [15], being a questionnaire designed to quantify the quality of life of patients attending otology clinics, and the Otology Questionnaire Amsterdam (OQUA) [8]. The OQUA is a generic otology questionnaire designed to evaluate the severity of ear complaints and their impact on patients’ lives. It possesses the unique quality that it can be used in ear patients and is designed to measure the change of symptoms during the course of the disease or treatment.

### Tinnitus

A wide array of tinnitus questionnaires exist designed to capture all domains that might be negatively affected by tinnitus in everyday life. Systematic reviews on tinnitus questionnaires usually focus on measures of health-related quality of life, reporting results limited to only the major self-report questionnaires [16–20]. All these reviews on tinnitus questionnaires present additional information on questionnaire characteristics (usually: number of items, score range, and (sub)scales)) or even some psychometric characteristics (e.g., internal consistency, test-retest reliability). From these reviews, all except two questionnaires were identified in our search. In addition to the questionnaires identified in these 5 review studies, we have identified an additional 12 tinnitus questionnaires.

### Vertigo

Reviews on vertigo in vestibular disease identified a total 15 questionnaires [21, 22]. All questionnaires were encountered in our results. Most vertigo questionnaires that we have identified were qualified as non-specific vertigo questionnaires (*n*=14). These questionnaires were non-disease-specific, nor did they adhere to a specific situation in which vertigo is provoked. Disease-specific questionnaires were most often specific for BPPD. This is consistent with BPPD as the primary cause of (vestibular) vertigo.

We have consulted two bibliographic databases for our search, which is considered a minimum. Nonetheless, a total number of 13,345 unique records were reviewed. This was the maximum capacity for our resources and is the highest number we have encountered during literature search in any systematic review in this field. All well-established questionnaires (e.g., Tinnitus Handicap Inventory [23], Dizziness Handicap Inventory [24], Abbreviated Profile of Hearing Aid Benefit [25]) were present in our results. In order to guarantee quality, our methods were in line with internationally recommended standards [PRISMA (Additional file 1), COSMIN]. This concerns the construction of our search syntax, the involvement of a medical information specialist (JCFK), the consultation of at least the Embase and MEDLINE databases, and at least two reviewers at every critical stage of the process with the possibility to discuss selection difficulties in an expert team [26].

The results of this study will provide a starting point for future research (e.g., regarding the psychometric properties of the questionnaires). The methodological quality of the development and validation of each included questionnaire and the quality of the measurement properties (see Additional file 3) of each questionnaire is a vast amount of data which will be assessed in forthcoming studies. This assessment will be done according to the COSMIN methodology for systematic reviews of patient-reported outcome measures [26]. This constitutes the use of the COSMIN Risk of Bias checklist for the assessment of the methodological quality of the studies and the measurement properties of the included questionnaires. Furthermore, measurement properties will be rated against the updated criteria for good measurement properties. The modified Grading of Recommendations Assessment, Development and Evaluation (GRADE) approach will be used for grading the quality of evidence of the reported outcomes on measurement properties.

## Conclusions

The presented work has systematically organized the existing questionnaires in otology. Clinicians and researchers benefit from the presented comprehensive overview of questionnaires as this enhances questionnaire selection. The database search performed in this systematic mapping review is the largest any study on this topic has assessed. Nearly every otology questionnaire identified in previously performed (systematic) reviews is included in this overview. Some suggestions for future studies on questionnaire measurement properties are proposed to facilitate an evidence-based questionnaire selection process.

## Supplementary Information

**Additional file 1.** PRISMA Checklist

**Additional file 2.** Search strategy for PubMed (26 August 2019)

**Additional file 3.** Patient-reported outcomes (PRO)-specific checklist

**Additional file 4.** Tinnitus questionnaires

**Additional file 5.** Vertigo questionnaires

**Additional file 6.** Hearing loss questionnaires

**Additional file 7.** Multiple complaint questionnaires

## Data Availability

All data generated or analyses during this study are included in this published article and its supplementary information files.

## Abbreviations

PROM: Patient-reported outcome measure
PRISMA: Preferred Reporting Items for Systematic reviews and Meta-Analyses for Protocols
PROSPERO: International Prospective Register of Systematic Reviews
KV: Koen Viergever
JCFK: Johannes CF Ket
QoL: Quality of life
HR-QoL: Health-related quality of life
EB: Els Bruinewoud
PRO: Patient-reported outcome
ERIQA: European Regulatory Issues on Quality of Life Assessment Working Group
COQOL: Cambridge Otology Quality of Life Questionnaire
OQUA: Otology Questionnaire Amsterdam
PANQOL: Penn Acoustic Neuroma Quality-of-Life
GRADE: Grading of Recommendations Assessment, Development and Evaluation

## References

[1] De Vet HCW, Terwee CB, Mokkink LB, Knol DL (2011). Measurement in medicine.

[2] Bess FH, Lichtenstein MJ, Logan SA (1990). Making hearing impairment functionally relevant: linkages with hearing disability and handicap. Acta Otolaryngol Suppl.

[3] Mulrow CD, Aguilar C, Endicott JE, Tuley MR, Velez R, Charlip WS (1990). Quality-of-life changes and hearing impairment. A randomized trial. Ann Intern Med.

[4] Saunders JE, Rankin Z, Noonan KY (2018). Otolaryngology and the global burden of disease. Otolaryngol Clin North Am.

[5] Granberg S, Dahlstrom J, Moller C, Kahari K, Danermark B (2014). The ICF core sets for hearing loss--researcher perspective. Part I: systematic review of outcome measures identified in audiological research. Int J Audiol.

[6] Akeroyd MA, Wright-Whyte K, Holman JA, Whitmer WM (2015). A comprehensive survey of hearing questionnaires: how many are there, what do they measure, and how have they been validated?. Trials.

[7] Bentler RA, Kramer SE (2000). Guidelines for choosing a self-report outcome measure. Ear Hear.

[8] Bruinewoud EM, Kraak JT, van Leeuwen LM, Kramer SE, Merkus P (2018). The Otology Questionnaire Amsterdam: a generic patient reported outcome measure about the severity and impact of ear complaints. A cross-sectional study on the development of this questionnaire. Clin Otolaryngol.

[9] Liberati A, Altman DG, Tetzlaff J, Mulrow C, Gotzsche PC, Ioannidis JP (2009). The PRISMA statement for reporting systematic reviews and meta-analyses of studies that evaluate healthcare interventions: explanation and elaboration. BMJ.

[10] Morgan A, Hickson L, Worrall L (2002). The impact of hearing impairment on quality of life of older people. Asia Pac J Speech Lang Hear.

[11] Chassany O, Sagnier P, Marquis P, Fullerton S, Aaronson N (2002). Patient-reported outcomes: the example of health-related quality of life—a European Guidance Document for the Improved Integration of Health-Related Quality of Life Assessment in the Drug Regulatory Process. Drug Inform J.

[12] Barker F, MacKenzie E, Elliott L, de Lusignan S (2015). Outcome measurement in adult auditory rehabilitation: a scoping review of measures used in randomized controlled trials. Ear Hear.

[13] Beach SR, Arias I (1983). Assessment of perceptual discrepancy: utility of the primary communication inventory. Fam Process.

[14] Kaplan H, Bally S, Brandt F, Busacco D, Pray J (1997). Communication Scale for Older Adults (CSOA). J Am Acad Audiol.

[15] Martin TP, Moualed D, Paul A, Ronan N, Tysome JR, Donnelly NP (2015). The Cambridge Otology Quality of Life Questionnaire: an otology-specific patient-recorded outcome measure. A paper describing the instrument design and a report of preliminary reliability and validity. Clin Otolaryngol.

[16] Meikle MB, Stewart BJ, Griest SE, Martin WH, Henry JA, Abrams HB (2007). Assessment of tinnitus: measurement of treatment outcomes. Prog Brain Res.

[17] Hall DA, Haider H, Szczepek AJ, Lau P, Rabau S, Jones-Diette J (2016). Systematic review of outcome domains and instruments used in clinical trials of tinnitus treatments in adults. Trials.

[18] Kamalski DM, Hoekstra CE, van Zanten BG, Grolman W, Rovers MM (2010). Measuring disease-specific health-related quality of life to evaluate treatment outcomes in tinnitus patients: a systematic review. Otolaryngol Head Neck Surg.

[19] Newman CW, Sandridge SA, Jacobson GP (2014). Assessing outcomes of tinnitus intervention. J Am Acad Audiol.

[20] Langguth B (2011). A review of tinnitus symptoms beyond ‘ringing in the ears’: a call to action. Curr Med Res Opin.

[21] Duracinsky M, Mosnier I, Bouccara D, Sterkers O, Chassany O (2007). Literature review of questionnaires assessing vertigo and dizziness, and their impact on patients’ quality of life. Value Health.

[22] Stewart VM, Mendis MD, Low CN (2018). A systematic review of patient-reported measures associated with vestibular dysfunction. Laryngoscope.

[23] Newman CW, Jacobson GP, Spitzer JB (1996). Development of the tinnitus handicap inventory. Arch Otolaryngol Head Neck Surg.

[24] Jacobson GP, Newman CW (1990). The development of the dizziness handicap inventory. Arch Otolaryngol Head Neck Surg.

[25] Cox RM, Alexander GC (1995). The abbreviated profile of hearing aid benefit. Ear Hear.

[26] Prinsen CAC, Mokkink LB, Bouter LM, Alonso J, Patrick DL, de Vet HCW (2018). COSMIN guideline for systematic reviews of patient-reported outcome measures. Qual Life Res.

