# Supplementary material for: Questionnaires in otology: a systematic mapping review
Source: Syst Rev. 2021 Apr 20;10:119. doi: 10.1186/s13643-021-01659-9 (PMC8059288; doi:10.1186/s13643-021-01659-9)
Supplement: Supplementary file 2 — Additional file 2. Search strategy for PubMed (26 August 2019) [file 13643_2021_1659_MOESM2_ESM.docx]

ADDITIONAL FILE 2 - SEARCH STRATEGY

**Search strategy for PubMed (26 August 2019)**

[Mesh] = Medical subject headings

[tiab] = words in title OR abstract

[mh] = MeSH Headings

| **Search** | **Query** | **Items found** |
| --- | --- | --- |
| **#5** | #4 NOT (("Adolescent"[Mesh] OR "Child"[Mesh] OR "Infant"[Mesh] OR adolescen*[tiab] OR child*[tiab] OR schoolchild*[tiab] OR infant*[tiab] OR girl*[tiab] OR boy*[tiab] OR teen[tiab] OR teens[tiab] OR teenager*[tiab] OR youth*[tiab] OR pediatr*[tiab] OR paediatr*[tiab] OR puber*[tiab]) NOT ("Adult"[Mesh] OR adult*[tiab] OR man[tiab] OR men[tiab] OR woman[tiab] OR women[tiab])) | **8,493** |
| **#4** | #3 NOT (animals[mh] NOT humans[mh]) | **12,045** |
| **#3** | #1 AND #2 | **12,097** |
| **#2** | "Earache"[MeSH] OR "Hearing Loss"[Mesh] OR "Hyperacusis"[Mesh] OR “Tinnitus"[MeSH] OR otalgi*[tiab] OR “aural discharge”[tiab] OR otorrhea[tiab] OR otorrea[tiab] OR “ear discharge"[tiab] OR earache*[tiab] OR hearing loss*[tiab] OR hypoacus*[tiab] OR hearing impairment*[tiab] OR deafness*[tiab] OR dysacus*[tiab] OR hyperacus* [tiab] OR (("Ear"[Mesh] OR "Ear Diseases"[Mesh] OR ear[tiab] OR ears[tiab] OR labyrinth*[tiab] OR cochlea*[tiab] OR endolymphat*[tiab] OR vestibular*[tiab] OR tympan*[tiab] OR otitis[tiab] OR semicircular*[tiab]) AND ("Pain"[Mesh] OR vertigo[Mesh] OR "Dizziness"[Mesh] OR “Taste”[MeSH] OR “Taste Perception”[MeSH] OR ageusia[Mesh] OR dysgeusia[Mesh] OR "Taste Disorders"[Mesh] OR “Pruritus”[MeSH] OR prurigo[MeSH] OR pain[tiab] OR vertigo*[tiab] OR dizziness*[tiab] OR tinnitus[tiab] OR taste*[tiab] OR tasting[tiab] OR ageusi*[tiab] OR dysgeusi*[tiab] OR “taste disorder”[tiab] OR gustator*[tiab] OR pruritus[tiab] OR prurigo*[tiab] OR itch*[tiab])) | **117,979** |
| **#1** | "Questionnaires"[Mesh] OR questionnaire*[tiab] | **1,199,134** |

**Search strategy for Embase.com (26 August 2019)**

/exp = EMtree keyword with explosion

/de = EMtree keyword without explosion

:ti,ab = words in title or abstract

/lim = limit

| **No.** | **Query** | **Results** |
| --- | --- | --- |
| **#5** | #4 NOT (('adolescent'/exp OR 'child'/exp OR adolescent*:ti,ab OR child*:ti,ab OR schoolchild*:ti,ab OR infant*:ti,ab OR girl*:ti,ab OR boy*:ti,ab OR teen:ti,ab OR teens:ti,ab OR teenager*:ti,ab OR youth*:ti,ab OR pediatr*:ti,ab OR paediatr*:ti,ab OR puber*:ti,ab) NOT ('adult'/exp OR 'aged'/exp OR 'middle aged'/exp OR adult*:ti,ab OR man:ti,ab OR men:ti,ab OR woman:ti,ab OR women:ti,ab)) | **7,882** |
| **#4** | #3 NOT ([animals]/lim NOT [humans]/lim) | **9,406** |
| **#3** | #1 AND #2 | **9,438** |
| **#2** | 'otalgia'/exp OR 'hearing impairment'/exp OR 'hearing disorder'/exp OR otalgi*:ab,ti OR 'aural discharge':ab,ti OR otorrhea:ab,ti OR otorrea:ab,ti OR 'ear discharge':ab,ti OR earache*:ab,ti OR ((hearing NEAR/3 (loss* OR impairment*)):ab,ti) OR hypoacus*:ab,ti OR deafness*:ab,ti OR dysacus*:ab,ti OR hyperacus*:ab,ti OR (('ear'/exp OR 'ear disease'/exp OR ear:ab,ti OR ears:ab,ti OR labyrinth*:ab,ti OR cochlea*:ab,ti OR endolymphat*:ab,ti OR vestibular*:ab,ti OR tympan*:ab,ti OR otitis:ab,ti OR semicircular*:ab,ti) AND ('pain'/exp OR 'vertigo'/exp OR 'dizziness'/exp OR 'taste disorder'/exp OR 'vestibular disorder'/exp OR 'taste'/exp OR 'pruritus'/exp OR pain:ab,ti OR vertigo*:ab,ti OR dizziness*:ab,ti OR tinnitus:ab,ti OR taste*:ab,ti OR tasting:ab,ti OR ageusi*:ab,ti OR dysgeusi*:ab,ti OR ((taste NEAR/3 disorder*):ab,ti) OR gustator*:ab,ti OR pruritus:ab,ti OR prurigo*:ab,ti OR itch*:ab,ti)) | **199,688** |
| **#1** | 'questionnaire'/de OR 'structured questionnaire'/exp OR questionnaire*:ab,ti | **861,064** |
