# Supplementary material for: Questionnaires in otology: a systematic mapping review
Source: Syst Rev. 2021 Apr 20;10:119. doi: 10.1186/s13643-021-01659-9 (PMC8059288; doi:10.1186/s13643-021-01659-9)
Supplement: Supplementary file 3 — Additional file 3. Patient-reported outcomes (PRO)-specific checklist [file 13643_2021_1659_MOESM3_ESM.docx]

ADDITIONAL FILE 3 - PATIENT-REPORTED OUTCOMES (PRO)-SPECIFIC CHECKLIST.

| Patient-Reported Outcomes (PRO)-specific checklist |
| --- |
| 1. Item generation and conceptual model underlying the development of the questionnaire. Level of patients’ input. |
| 2. Description of the construct measured by the questionnaire. |
| 3. Description and adequacy of the population involved in the different steps of validation. |
| 4. Size of the population involved in the different steps of validation. |
| 5. Description of the questionnaire (author, publication date, number of items, (sub)scales). |
| 6. Scaling (response options) and scoring (including score ranges). |
| 7. Recall period (period to which answers must relate). |
| 8. Item reduction process (may involve distribution of response options, content validity, expert opinion, and  8 psychometric analyses: i.e., factor analysis). |
| 9. Internal consistency (level of correlation of the items in a dimension). |
| 10. Test-retest (stability of scores over time when patients’ condition is considered stable). |
| 11. Content validity (items and response options that are relevant and comprehensive of the dimensions). |
| 12. Structural validity (factor analysis or similar to support the hypothesized scale structure: i.e., the combinations of  items into dimensions). |
| 13. Discriminant validity (capacity of the questionnaire to discriminate the patients according to certain  characteristics, e.g., severity of the disease). |
| 14. Convergent validity (correlations of the questionnaire with a scale known as reference or which is  supposed to measure more or less similar concepts). |
| 15. Predictive validity (future evolution of the disease can be predicted by score changes of the questionnaire). |
| 16. Responsiveness (ability to detect changes). |

Patient-Reported Outcomes (PRO)-specific checklist derived from the European Regulatory Issues on Quality of Life Assessment Working Group (ERIQA) for the systematic appraisal of the validation of PROM’s. This checklist was used for the extraction of questionnaire characteristics used in this study and for forthcoming studies on the measurement properties of the identified questionnaires.
