# Supplementary figures and images for: Questionnaires in otology: a systematic mapping review
Source: Syst Rev. 2021 Apr 20;10:119. doi: 10.1186/s13643-021-01659-9 (PMC8059288; doi:10.1186/s13643-021-01659-9)

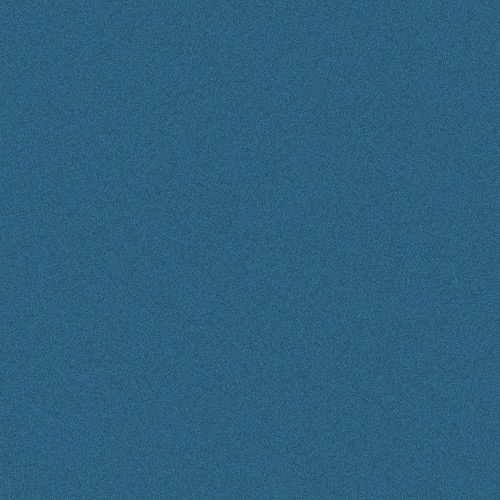

Supplement: Supplementary file 4 — Additional file 4. Tinnitus questionnaires [file 13643_2021_1659_MOESM4_ESM.docx › Data/PresetImageFill0-15.jpg]

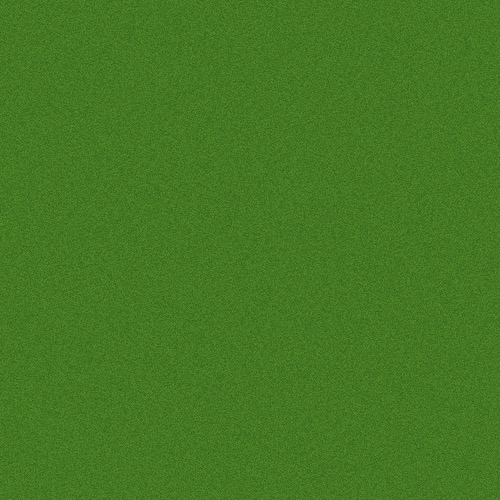

Supplement: Supplementary file 4 — Additional file 4. Tinnitus questionnaires [file 13643_2021_1659_MOESM4_ESM.docx › Data/PresetImageFill1-16.jpg]

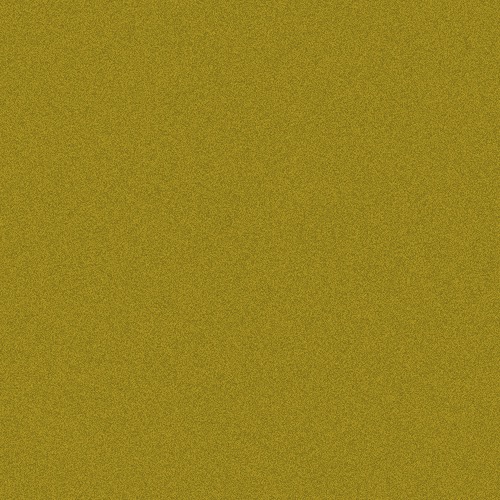

Supplement: Supplementary file 4 — Additional file 4. Tinnitus questionnaires [file 13643_2021_1659_MOESM4_ESM.docx › Data/PresetImageFill2-17.jpg]

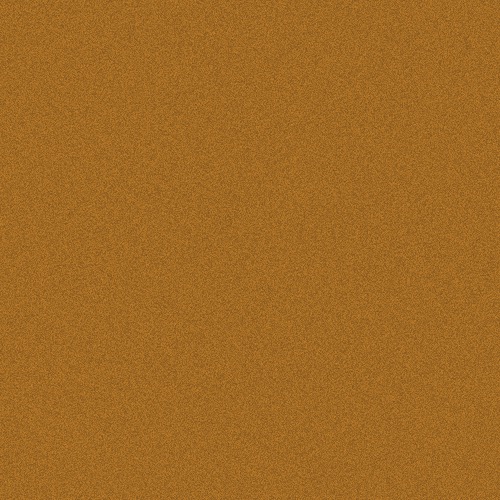

Supplement: Supplementary file 4 — Additional file 4. Tinnitus questionnaires [file 13643_2021_1659_MOESM4_ESM.docx › Data/PresetImageFill3-18.jpg]

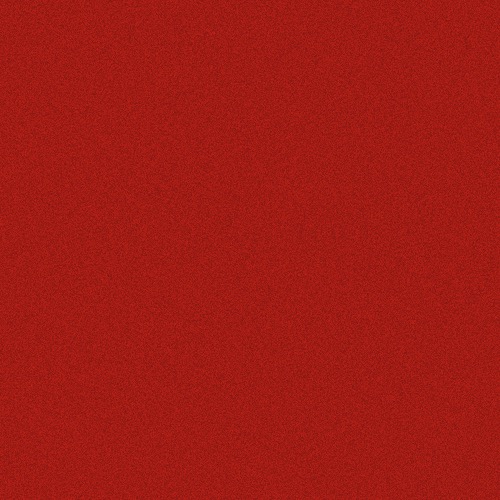

Supplement: Supplementary file 4 — Additional file 4. Tinnitus questionnaires [file 13643_2021_1659_MOESM4_ESM.docx › Data/PresetImageFill4-19.jpg]

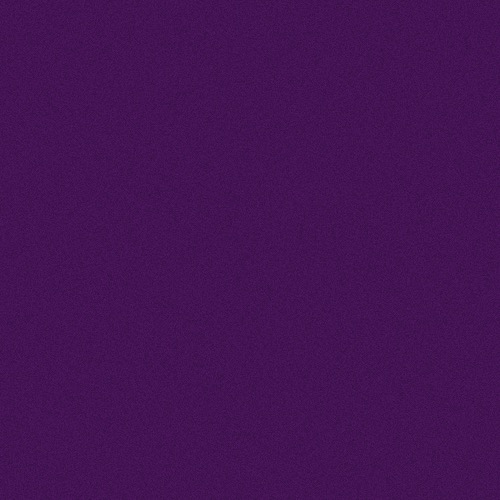

Supplement: Supplementary file 4 — Additional file 4. Tinnitus questionnaires [file 13643_2021_1659_MOESM4_ESM.docx › Data/PresetImageFill5-20.jpg]

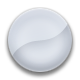

Supplement: Supplementary file 4 — Additional file 4. Tinnitus questionnaires [file 13643_2021_1659_MOESM4_ESM.docx › Data/bullet_gbutton_gray-21.png]

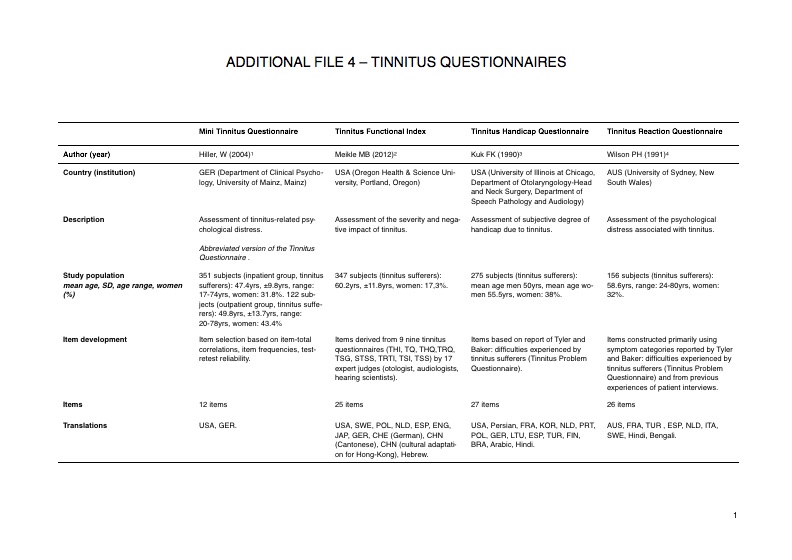

Supplement: Supplementary file 4 — Additional file 4. Tinnitus questionnaires [file 13643_2021_1659_MOESM4_ESM.docx › preview.jpg]

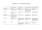

Supplement: Supplementary file 4 — Additional file 4. Tinnitus questionnaires [file 13643_2021_1659_MOESM4_ESM.docx › preview-micro.jpg]

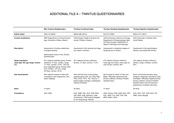

Supplement: Supplementary file 4 — Additional file 4. Tinnitus questionnaires [file 13643_2021_1659_MOESM4_ESM.docx › preview-web.jpg]

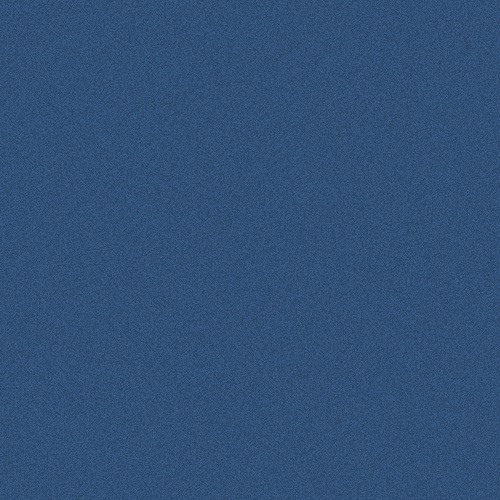

Supplement: Supplementary file 5 — Additional file 5. Vertigo questionnaires [file 13643_2021_1659_MOESM5_ESM.docx › Data/PresetImageFill0-15.jpg]

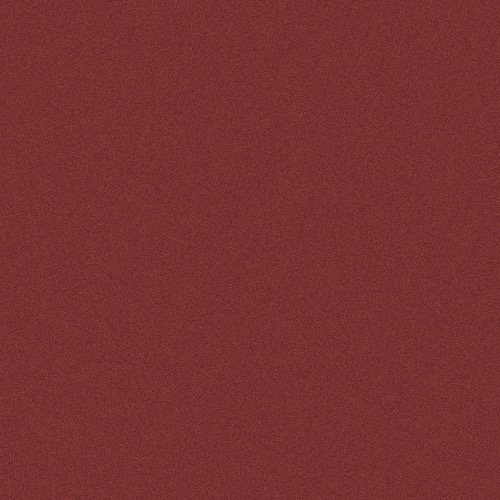

Supplement: Supplementary file 5 — Additional file 5. Vertigo questionnaires [file 13643_2021_1659_MOESM5_ESM.docx › Data/PresetImageFill1-16.jpg]

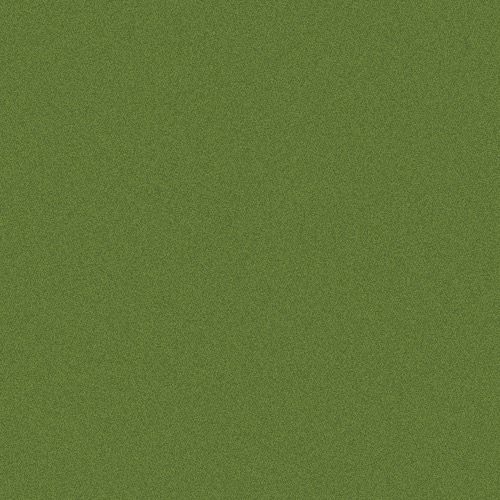

Supplement: Supplementary file 5 — Additional file 5. Vertigo questionnaires [file 13643_2021_1659_MOESM5_ESM.docx › Data/PresetImageFill2-17.jpg]

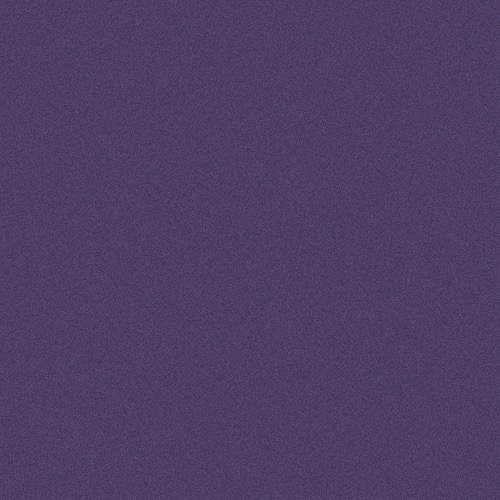

Supplement: Supplementary file 5 — Additional file 5. Vertigo questionnaires [file 13643_2021_1659_MOESM5_ESM.docx › Data/PresetImageFill3-18.jpg]

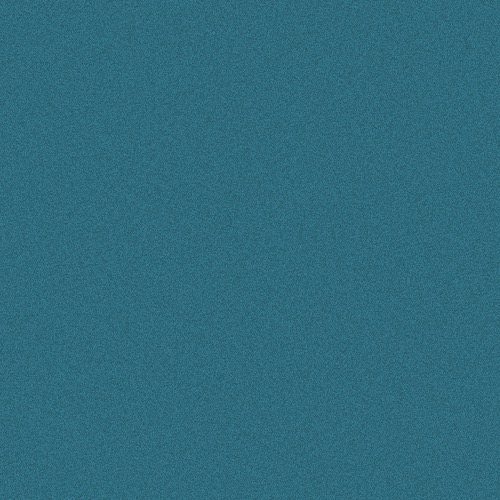

Supplement: Supplementary file 5 — Additional file 5. Vertigo questionnaires [file 13643_2021_1659_MOESM5_ESM.docx › Data/PresetImageFill4-19.jpg]

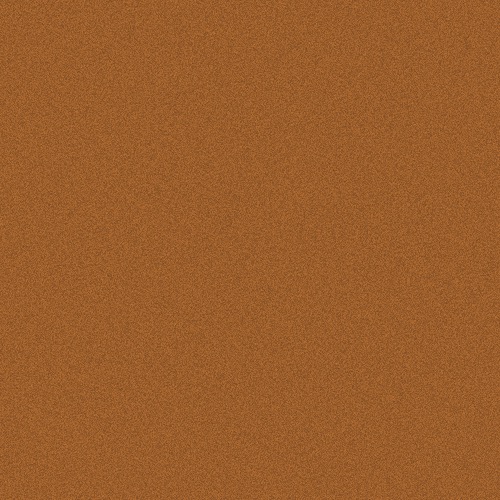

Supplement: Supplementary file 5 — Additional file 5. Vertigo questionnaires [file 13643_2021_1659_MOESM5_ESM.docx › Data/PresetImageFill5-20.jpg]

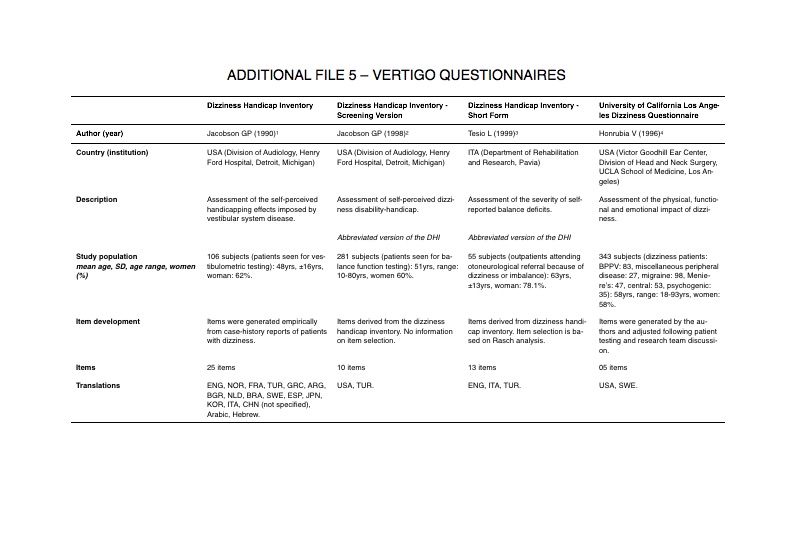

Supplement: Supplementary file 5 — Additional file 5. Vertigo questionnaires [file 13643_2021_1659_MOESM5_ESM.docx › preview.jpg]

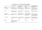

Supplement: Supplementary file 5 — Additional file 5. Vertigo questionnaires [file 13643_2021_1659_MOESM5_ESM.docx › preview-micro.jpg]

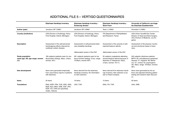

Supplement: Supplementary file 5 — Additional file 5. Vertigo questionnaires [file 13643_2021_1659_MOESM5_ESM.docx › preview-web.jpg]
