# Supplementary material for: Questionnaires in otology: a systematic mapping review
Source: Syst Rev. 2021 Apr 20;10:119. doi: 10.1186/s13643-021-01659-9 (PMC8059288; doi:10.1186/s13643-021-01659-9)
Supplement: Supplementary file 6 — Additional file 6. Hearing loss questionnaires [file 13643_2021_1659_MOESM6_ESM.docx]

ADDITIONAL FILE 6 - HEARING LOSS QUESTIONNAIRES

|  | **Hyperacusis Questionnaire** | **Hyperacusis Questionnaire - Short Version** | **Multiple-Activity Scale for Hyperacusis** | **Abbreviated Profile of Hearing Aid Benefit** |
| --- | --- | --- | --- | --- |
| **Author (year)** | Khalfa S (2002)^1^ | Tortorella F (2017)^2^ | Dauman R (2005)^3^ | Cox RM (1995)^4^ |
| **Country (institution)** | FRA (Neurosciences and Sensory Systems, Lyon) | ITA (Department of Applied Clinical Sciences and Biotechnology, L’Aquila University, L’Aquila) | FRA (Tinnitus Clinic, Department of Otolaryngology*/Head and Neck Surgery, Unit of Audiology, University Hospital of Bordeaux, University Victor Segalen, Bordeaux) | USA (The University of Memphis, and Veterans Affairs Medical Center, Memphis, Tennessee) |
| **Description** | Quantification and characterization of hyperacusis over three major dimensions: attentional, social and emotional. | Identification of hyperacusis. | Assessment of hyperacusis annoyance. | Quantifying the disability associated with a hearing loss and the reduction of disability that is achieved with a hearing aid. |
| **Study population**  ***mean age, SD, age range, women (%)*** | 201 subjects (random persons from the general population responding to an advertisement for subject recruitment): 28.4yrs, ±13.24yrs, range 17-72yrs, women: 65.6%. | 117 subjects (tinnitus patients): 53yrs, range 23-82yrs, women: 42%. | 249 subjects (tinnitus patients suffering from continuous tinnitus, no CI patients and no profoundly deaf patients who did not use hearing aids): males (52yrs, ±13.3yrs) and females (54 yrs, ±14.3yrs), women: 42,2%. | 128 subjects ((elderly) hearing-impaired using conventional analog hearing aids): 68yrs, range: 30-87yrs, women: 29.6%. |
| **Item development** | Items are generated based on literature findings. | Items originate from the original HQ by Khalfa, selected based on odd’s ratio’s estimates for hyperacusis according to the item responses. | No description of item generation. | Items are derived from the Profile of Hearing Aid Benefit. Item selection was based on item-total correlations on the items of 4 selected subscales. |
| **Items** | 14 items | 06 items | 14 items | 24 items |
| **Translations** | ENG, FRA, ITA, NLD, JPN, TUR. | ENG, ITA. | ENG, FRA, NLD. | USA,, GER, KOR, NOR, ITA, NLD, FRA, ESP, POL, PRT SWE, JPN, SGP, CHN (Hong-Kong), CHN (Cantonese), CAN (French). |

|  | **Satisfaction with Amplification in Daily Life** | **Effectiveness of Auditory Rehabilitation scale** | **International Outcome Inventory for Hearing Aids** | **Auditory Behaviour Questionnaire** |
| --- | --- | --- | --- | --- |
| **Author (year)** | Cox RM (1999)^5^ | Yueh B (2005)^6^ | Cox R (2000)^7^ | Diges I (2017)^8^ |
| **Country (institution)** | USA (The University of Memphis, and Department of Veterans Affairs Medical Center, Tennessee) | USA (Health Services Research and Development Service, University of Washington, Seattle) | USA (University of Memphis, School of Audiology and Speech-Language Pathology, Memphis, Tennessee) | ESP (ACURE-Tinnitus and Hyperacusis Clinic, Madrid) |
| **Description** | Quantifying satisfaction with hearing aids. | Assessment of the effectiveness of amplification in the treatment of sensorineural hearing loss. | Assessment of the effectiveness of hearing aid treatments. | Assessment of the auditory processing difficulties associated to hearing impaired subjects. |
| **Study population**  ***mean age, SD, age range, women (%)*** | 257 subjects (hearing aid users): range: 60-89yrs, women: 23.34%. | 78 subjects (adult patients presenting for bilateral hearing aid evaluations): 72.1yrs, women: 19%. | 172 subjects (hearing aid owners): 72yrs, range: 26-98yrs, women: 42%. | 305 subjects (patients attending an audiologic clinic): 174 men (44yrs, ±14 yrs) and 131 women (47yrs, ±14 yrs), women: 42.95%. |
| **Item development** | Items are based on literature study and patient interviews. | Items are generated based on patient input (interview, diary, and open-ended questionnaire data), literature study, and authors’ clinical expertise. | No description of item generation. | Item generation is guided by clinical expertise from ENT specialists, audiologist, and psychologists and based on patient feedback. |
| **Items** | 15 items | 23 items | 07 items | 25 items |
| **Translations** | USA, PRT, BRA, GER, Persian, Hebrew. | USA, CHL (Spanish). | USA, SWE, NLD, GER, DNK, FIN, FRA, GER, GRK, HUN, ITA JPN, KOR, MYS, NOR, POL, BRA, PRT, RUS, SRB,, SVK, SVN, ESP, TUR, WAL, CHN (simplified), CHN (traditional), Arabic, Sinhalese, Hebrew, Kannada. | ENG, ESP. |

|  | **Profile of Hearing Aid Performance** | **Modified Abbreviated Profile of Hearing Aid Performance** | **Profile of Hearing Aid Benefit** | **Self-assessment of Hearing Screening of the Elderly** |
| --- | --- | --- | --- | --- |
| **Author (year)** | Cox RM (1990)^9^ | Purdy SC (1998)^10^ | Cox RM (1991)^11^ | Kim G (2016)^12^ |
| **Country (institution)** | USA (Department of Audiology and Speech Pathology, Memphis State University, and Veteran's Administration Medical Center, Memphis, Tennessee) | NZL (Audiology Section, Department of Physiology, Faculty of Medicine and Health Science, The University of Auckland, Auckland, New Zealand) | USA (Memphis State University, and Department of Veterans Affairs Medical Center, Memphis, Tennessee) | KOR (Department of Speech Pathology and Audiology, Hallym University Graduate School, Chuncheon, Republic of Korea) |
| **Description** | Assessment of hearing aid performance in everyday life. | Assessment of hearing aid performance in everyday life.  *Abbreviated version of the Profile of Hearing Aid Performance* | Assessment of hearing aid benefit. | Assessment of the degree of hearing loss in the elderly. |
| **Study population**  ***mean age, SD, age range, women (%)*** | 225 subjects (hearing aid wearers). | 79 subjects, 2 groups (uni- and bilateral hearing aid wearers): 67 subjects: 64.9yrs, ±12.7yrs, range: 26-88yrs. 12 subjects: 59yrs, ±9.1yrs, range: 46-74yrs. | 42 patients (hearing aid wearers): 69yrs. | 83 subjects (elderly members of community): 77.33yrs, range: 68–84yrs, women: 34,93%. |
| **Item development** | Items are mainly derived from other scales: Hearing Aid Performance Inventory and the Communication Profile of the Hearing Impaired). | All items are derived from the Abbreviated Profile of the Hearing Impaired. | Items are derived form the Profile of Hearing Aid Performance. | Items are extracted from eleven questionnaires (eg, APHAP, COSI, GHABP, HHIA, HHIE, IOI-HA, KESHH (Korean Evaluation Scale for Hearing Handicap), PHAB, SADL, SSQ). |
| **Items** | 66 items | 22 items | 66 items | 20 items |
| **Translations** | USA. | USA. | USA. | ENG, KOR. |

|  | **Profile of Aided Loudness** | **Measure of Audiologic Rehabilitation Self-Efficacy for Hearing Aids** | **Listening Self Efficacy Questionnaire** | **Device-Oriented Subjective Outcome Scale** |
| --- | --- | --- | --- | --- |
| **Author (year)** | Palmer CV (1999)^13^ | West RL (2007)^14^ | Smith SL (2011)^15^ | Cox RM (2014)^16^ |
| **Country (institution)** | USA (University of Pittsburgh, Section of Audiology, Department of Ear Nose and Throat) | USA (Department of Psychology, University of Florida, Gainesville, Florida) | USA (Department of Audiology and Speech-Language Pathology, East Tennessee State University, Johnson City, Tennessee) | USA (University of Memphis, Memphis, Tennessee) |
| **Description** | Assessment of hearing aid fittings that have as a goal to return normal loudness perception. | Assessment of confidence in one’s ability to be a successful hearing-aid user. | Assessment of the beliefs (i.e. confidence) listeners have in their capabilities to plan and execute the actions needed to understand speech in a variety of listening situations. | Assessment of hearing aid device-oriented outcomes. |
| **Study population**  ***mean age, SD, age range, women (%)*** | 41 subjects (normally hearing subjects): range: 20-65yrs. | 211 subjects, 2 groups (new hearing aid users (NHA) and experienced hearing aid users (EHA)): 83 subjects (NHA): 71.8yrs, ±8.3yrs, women: 0%. 128 subjects (EHA): 73.0yrs, ±8.4yrs, women: 0%. | 169 subjects (older untreated sensorineural hearing loss sufferers): 65.9yrs, ±6.7yrs, range: 55-85yrs, women: 1.7%. | 295 subjects (uni- and bilateral hearing aid wearers): 72yrs, ±10.7yrs, range: 21-94yrs, women: 40%. |
| **Item development** | No description of item generation. | Items are generated based on literature study. | Items are generated based on questionnaire content (APHAB, HHIE, MARS-HA, SSQ) and commonly heard patient complaints. | Items are generated by authors experienced in the field of hearing questionnaire (APHAB and SADL) and selected based on audiologist and patient feedback. |
| **Items** | 12 items | 24 items | 18 items | 40 items |
| **Translations** | USA. | USA, CAN (French). | USA. | USA. |

|  | **Hearing Aid Satisfaction Questionnaire** | **Screening Test for Hearing Problems** | **Shortened/modified Hearing Measurement Scale** | **Hearing Handicap Inventory for the Elderly** |
| --- | --- | --- | --- | --- |
| **Author (year)** | Korkmaz MH (2016)^17^ | Demorest ME (2011)^18^ | Espmark AK (2002)^19^ | Ventry IM (1982)^20^ |
| **Country (institution)** | TUR (Department of Otolaryngology and Head and Neck Surgery, Faculty of Medicine, Yıldırım Beyazıt University, Ankara) | USA (University of Maryland, Baltimore County, Baltimore) | SWE (Department of Audiology, Goteborg University, Goteborg) | USA (Teachers College, Columbia University, New York, New York) |
| **Description** | Assessment patient compliance and satisfaction for hearing aids in hearing aid prescribed adults. | Assessment of communication  problems and psychosocial adjustment to hearing impairment. | Assessment of hearing handicap in everyday life.  *Abbreviated version of the Hearing Measurement Scale.* | Assessment of the effects of hearing impairment on the emotional and social adjustment of elderly people. |
| **Study population**  ***mean age, SD, age range, women (%)*** | 400 subjects (hearing aid wearers): 63.67yrs, ±12.12yrs, range: 39–89yrs, women: 42.75%. | 1319 subjects (patients attending a clinic for audiological assessment): 65.0yrs, ±15.4, range: 16– 97yrs, women: 44.2%. | 154 subjects (elderly living at home): range (men): 70-90yrs, range (women): 70-91yrs, women: 62%. | 100 subjects (community-based individuals): 75yrs, range: 65-92yrs, women: 52%. |
| **Item development** | No description of item generation. | Items are derived from the Communication Profile for the Hearing Impaired. | Items are derived from the Hearing Measurement Scale. No description of item selection. | Item generation was performed by an expert group of 5 audiologists. |
| **Items** | 10 items | 20 items | 20 items | 25 items |
| **Translations** | ENG, TUR. | USA. | AUS, SWE. | USA, SWE, PRT, BRA, JPN, GER, KOR, CHN (Mandarin), Yupik, Yoruba. |

|  | **Hearing Handicap Inventory for the Elderly - Screening/shortened version** | **Hearing Handicap Inventory for Adults** | **Hearing Handicap Inventory for Adults - Screening Version** | **Hearing Measurement Scale** |
| --- | --- | --- | --- | --- |
| **Author (year)** | Lichtenstein MJ (1988)^21^ | Newman CW (1990)^22^ | Newman CW (1991)^23^ | Noble WG (1979)^24^ |
| **Country (institution)** | USA (Division of General Internal Medicine, Vanderbilt University Medical Center, Nashville, Tennessee) | USA (Division of Audiology, Henry Ford Hospital, Detroit, Michigan) | USA (Division of Audiology, Henry Ford Hospital, Detroit, Michigan) | AUS (Department of Psychology, University of New England, Armidale, New South Wales) |
| **Description** | Assessment of the effects of hearing impairment on the emotional and social adjustment of elderly people.  *Abbreviated version of the Hearing Handicap Inventory for the Elderly.* | Assessment of the effects of hearing impairment on the emotional and social/situational adjustment of adults <65 yrs of age. | Assessment of the effects of hearing impairment on the emotional and social/situational adjustment of adults <65 yrs of age.  *Abbreviated version of the Hearing Handicap Inventory for Adults.* | Assessment of hearing handicap in everyday life. |
| **Study population**  ***mean age, SD, age range, women (%)*** | 178 subjects (patients over 65yrs old screened in primary care  practice for hearing loss): 74.2yrs, ±6.4yrs, women: 62.9%. | 67 subjects (adult outpatients presenting with normal or hearing or sensorineural hearing loss): 48.7yrs, ±12yrs, range: 18-64yrs, women: 34.3%. | 28 subjects (hearing impaired outpatients undergoing audiologic evaluation. Previous or current hearing aid users were excluded from participating): 44.2yrs, ±8.6yrs, range: 28-59yrs. | 23 subjects (patients with noise induced hearing loss): 48-50yrs (2 groups), ±10.4-11.9, women: 0%. |
| **Item development** | Items are derived from the Hearing Handicap Inventory for the Elderly. No description of item selection. | All items are originally from the HHIE, with 3 items being modified. | Items are derived from the HHIA. | Items originate from the original hearing measurement scale (interview form) and were reworded where necessary. |
| **Items** | 10 items | 25 items | 10 items | 42 items |
| **Translations** | USA, SWE, FIN,, CHN (Mandarin), BRA, JAP, SRB, NLD, TWN, IDN, Indian, Kannada, Arabic, MEX (Spanish), Spanish (Spanish speaking Mexican Americans). | USA, ITA, FRA, MYS, BRA, SWE, Indian, Kannada. | USA, JPN, PRT. | AUS, SWE, JPN, FIN. |

|  | **Amsterdam Inventory for Auditory Disability and Handicap** | **(modified) Amsterdam Inventory for Auditory Disability and Handicap** | **Hearing Handicap Scale** | **Communication Profile for the Hearing Impaired** |
| --- | --- | --- | --- | --- |
| **Author (year)** | Kramer SE (1995)^25^ | Meijer AG (2003)^26^ | High WS (1964)^27^ | Demorest ME (1987)^28^ |
| **Country (institution)** | NLD (Clinical Audiology, Department of Otolaryngology, University Hospital VU, Amsterdam) | NLD (Department of Otolaryngology, University Hospital Groningen) | USA (Noise Research Center) | USA (University of Maryland Baltimore County) |
| **Description** | Assessment of hearing disability in everyday life. | Assessment of hearing disability in everyday life. | Assessment of handicap resulting from hearing impairment. | Assessment of communication problems in hearing impaired persons. |
| **Study population**  ***mean age, SD, age range, women (%)*** | 274 subjects (hearing impaired, age <66yrs): 48.3yrs, range: 16-65yrs, women: 27.3%. | 94 subjects (patients at an ENT-department not wearing a hearing aid): 41yrs, range: 17-65yrs, women: 56.3%. | 50 subjects (patients with chronic hearing impairment): 48.8yrs, range:21-72yrs, women: 36%. | 433 subjects (patients attending the Army Audiology and Speech Center's Aural Rehabilitation Program: active-duty and retired military personnel who are issued hearing aids): range: 20-17yrs, women: 4%. |
| **Item development** | Items are generated from patient interviews/feedback, existing questionnaires (Hearing Measurement Scale, Hearing Handicap Scale, Hearing Disability Questionnaire, Hearing Disability and Handicap Scale (HDHS)) and clinical expertise. | Items are adapted from the original AIADH (i.e. reduced to only the A-part of each item). | Items are generated by authors revised after expert feedback (41 otologists and audiologists). | Items are generated by authors and clinical experts based on patient reports, clinical experience and existing questionnaires (Hearing Handicap Scale, the Hearing Measurement Scale, the Denver Scale of Communication Function, the Hearing Performance Inventory, and the Hearing Problem Inventory. |
| **Items** | 28 items | 28 items | 40 items | 145 items |
| **Translations** | ENG, NLD, ESP, SWE, BRA, POL, CHN (Hong-Kong). | ENG, NLD. | USA. | USA, NLD, SWE. |

|  | **Speech, Spatial and Qualities of Hearing Scale** | **Hearing Handicap Questionnaire** | **Abbreviated Speech Spatial and Qualities of Hearing Scale** | **Quantified Denver Scale of Communication Function** |
| --- | --- | --- | --- | --- |
| **Author (year)** | Gatehouse S (2004)^29^ | Gatehouse S (2004)^29^ | Noble W (2013)^30^ | Schow RL (1980)^31^ |
| **Country (institution)** | SCH (MRC Institute of Hearing Research, Glasgow) | SCH (MRC Institute of Hearing Research, Glasgow) | AUS (School of Behavioural, Cognitive and Social Sciences, University of New England) | USA (Health Services Research, Audie I. Murphy Memorial Veterans Administration Hospital, San Antonio, Texas) |
| **Description** | Assessment of auditory disability in speech, sound localization and other qualities (i.e. segregation of sounds, recognition, clarity/naturalness, and listening effort). | Assessment of hearing related handicap. | Assessment of auditory disability in speech, sound localization and other listening/sound qualities. Abbreviated version of the Speech, Spatial and Qualities of Hearing Scale. | Assessment of communication difficulties in adults with hearing impairment. |
| **Study population**  ***mean age, SD, age range, women (%)*** | 153 subjects (new referrals prior to hearing aid fitting): 71yrs, ±8.1yrs, women: 52.3%. | 153 subjects (new referrals prior to hearing aid fitting): 71yrs, ±8.1yrs, women: 52.3%. | 1220 subjects (data set of the MRC Institute of Hearing Research Scottish Section). | 50 subjects (individuals interested in determining their hearing status: normal hearing to hearing loss sufferers): 55.8yrs, range: 19-78yrs, women: 54%. |
| **Item development** | Items are generated by the authors. | Items are derived in part from items in the Hearing Disabilities and Handicaps Scale, and from items in the Glasgow Health Status Inventory, with adjustment of wording to ask specifically about effects of hearing. | Items are derived by expert consent from the SSQ, based on their experiences with the SSQ. | Items are derived from the Denver Scale of Communication Function. No description of adjustments. |
| **Items** | 49 items | 12 items | 12 items | 25 items |
| **Translations** | SCH, NLD, FRA, GER, Persian, RUS, POL, ESP, SWE, KOR, Arabic. | SCH (English), Kannada. | ENG, DNK, NLD. | USA. |

|  | **Short Quantified Denver Scale of Communication Function** | **Revised Quantified Denver Scale of Communication Function** | **Hearing Performance Inventory** | **Gothenburg Profile** |
| --- | --- | --- | --- | --- |
| **Author (year)** | Tuley MR (1990)^32^ | Tuley MR (1990)^32^ | Giolas TG (1979)^33^ | Ringdahl A (1998)^34^ |
| **Country (institution)** | USA (Health Services Research and Development Satellite Unit, Audie 1. Murphy Memorial Veterans’ Administration Hospital, San Antonio, Texas) | USA (Health Services Research and Development Satellite Unit, Audie 1. Murphy Memorial Veterans’ Administration Hospital, San Antonio, Texas) | USA (Department of Speech, U-85, University of Connecticut, Storrs, Connecticut) | SWE (Department of Audiology, Sahlgrens University Hospital) |
| **Description** | Assessment of self-isolation and communication function in elderly individuals with hearing loss. | Assessment of hearing handicap in elderly individuals. | Assessment of hearing performance in problem areas experienced in everyday listening. | Assessment of hearing disability and handicap in hearing aid wearers and hearing aid candidates. |
| **Study population**  ***mean age, SD, age range, women (%)*** | 238 subjects (elderly individuals (>64yrs) with and without hearing loss): 71.6yrs, ±5.5yrs, women: 1%. | 238 subjects (elderly individuals (>64yrs) with and without hearing loss): 71.6yrs, ±5.5yrs, women: 1%. | 220 subjects (hearing impaired people). | 919 subjects, 2 groups (new hearing aid candidates (NHA) and experienced hearing aid users (EHA), 598 subjects (NHA): 66 yrs, ±17.0; range 14-91yrs. 326 subjects (EHA): 71yrs, ±15.0yrs, range: 14-91yrs. |
| **Item development** | Items are derived from the Quantified Denver Scale of Communication Function. Item selection was performed on the basis of stepwise discriminant analysis. | Items are the same as in the original Quantified Denver Scale of Communication Function. | Items are generated by the authors, based on input from hearing impaired people, audiologist, normal hearing people, and literature. | Items are partly taken from the shortened Hearing Measurement Scale. |
| **Items** | 05 items | 25 items | 158 items | 20 items |
| **Translations** | USA. | USA. | USA, SWE. | ENG, SWE, GER, DNK, NLD. |

|  | **Hearing Disability Acceptance Questionnaire** | **Self Assessment of Communication** | **Hearing Self-Assessment Questionnaire** | **Performance Inventory for Profound and Severe Loss** |
| --- | --- | --- | --- | --- |
| **Author (year)** | Machaiah VKC (2014)^35^ | Schow RL (1982)^36^ | Bonetti L (2018)^37^ | Owens E (1988)^38^ |
| **Country (institution)** | ENG (Department of Vision and Hearing Sciences, Anglia Ruskin University, Cambridge) | USA (Department of Speech Pathology and Audiology, Idaho State University, Pocatello, Idaho) | HRV (Faculty of Education and Rehabilitation Sciences, University of Zagreb, Zagreb) | USA (University of California, San Francisco) |
| **Description** | Assessment of the acceptance of hearing disability. | Assessment of hearing disability in adults using hearing aids. | Identification of functional hearing difficulties. | Assessment of hearing performance in communicative situations of persons with profound and severe hearing losses. |
| **Study population**  ***mean age, SD, age range, women (%)*** | 90 subjects (self assessed hearing difficulty sufferers without hearing aids): 63.41yrs, ±10.49yrs, women: 50%. | 50 subjects (hearing and hearing-impaired individuals): range: 20-80yrs. | 112 subjects (outpatients at an ENT-clinic referred for audiological examination): 56.24yrs, ±12.92yrs, range: 24-88yrs, women: 42.85%. | 50 subjects (hearing aid users): 57yrs, range: 30-77yrs, women: 58%. |
| **Item development** | Items are based on the Tinnitus Acceptance Questionnaire: the word ‘tinnitus’ was replaced with  ‘hearing problem’ and the total number of items was reduced from 12 to 7 based on principal component analysis. | Item are generation based on all major aspects identified in existing self-assessment scales, especially the Hearing Performance Inventory. | Items are generated based on survey results among hearing impaired adults. | Items are generated from patient interviews and partly included (un)changed from the Revised Hearing Performance Inventory. |
| **Items** | 07 items | 10 items | 10 items | 74 items |
| **Translations** | ENG. | USA, TUR, BRA, Kannada. | ENG, HRV. | USA, NOR. |

|  | **Hearing Dependant Daily Activities Scale** | **Communication Self-Assessment Scale for Deaf Adults** | **Revised Hearing Performance Inventory** | **Reduced Speech, Spatial and Qualities of Hearing Scale** |
| --- | --- | --- | --- | --- |
| **Author (year)** | Hidalgo JL (2008)^39^ | Kaplan H (1991)^40^ | Lamb SH (1983)^41^ | Demeester K (2012)^42^ |
| **Country (institution)** | ESP (Family and Community Medicine, Faculty of Medicine, University of Castilla-La Mancha, Albacete) | USA (Department of Audiology and Speech/Language Pathology, Gallaudet University, Washington, DC) | USA (San Francisco State University, San Francisco, California) | BEL (Department of Otolaryngology, University (UA) and University Hospital of Antwerp (UZA)) |
| **Description** | Assessment of the impact of hearing loss on daily life in the elderly (>64 yrs). | Assessment of communication in deaf adults. | Assessment of hearing performance in problem areas experienced in everyday listening.  *Abbreviated version of the Hearing Performance Inventory* . | Assessment of hearing impairment. |
| **Study population**  ***mean age, SD, age range, women (%)*** | 1160 subjects (random selection of persons aged 65 or older registered in the National Health Care system): 73.3yrs, ±5.9yrs, range: 65-96yrs, women: 55.9%. | 290 subjects (deaf adults: severe or profound bilateral sensorineural hearing loss): 20.9yrs, women: 51.7%. | 354 subjects (hearing impaired subjects). | 235 subjects, 3 groups (normal hearing (NH), clinically normal hearing (CNH) and hearing impaired (HI): 103 subjects (NH): 19,5yrs, ±1,4yrs, range: 18-25yrs. 24 subjects (CNH): 62,9yrs, ±2,7yrs, range: 55-65yrs. 109 subjects (HI): 62,11yrs, ±5,4yrs, range: 55-65yrs. |
| **Item development** | Items are generated by the authors after review of existing questionnaires (e.g. HHIE) and literature. | Items are generated by authors on the basis of own expertise and existing scales, adjusted by patient and expert feedback. | Items originate from the Hearing Performance Inventory. Items were selected in part on a statistical basis and in part on clinical relevance. | Items originate from the SSQ. Item selection performed by cluster analyses and binary logistic regression analyses. |
| **Items** | 12 items | 115 items | 90 items | 05 items |
| **Translations** | ENG, ESP. | USA. | USA. | USA, BEL. |

|  | **Shortened Hearing Aid Performance Inventory** | **Social Isolation Measure** | **Social Participation and Restriction Questionnaire** | **Inventory of Hyperacusis Symptoms** |
| --- | --- | --- | --- | --- |
| **Author (year)** | Schum DJ (1992)^43^ | Hefffernan E (2019)^44^ | Hefffernan E (2018)^45^ | Greenberg B (2018)^46^ |
| **Country (institution)** | USA (Department of Otolaryngology, University of Iowa Hospital, Iowa City, Iowa) | ENG (National Institute for Health Research (NIHR) Nottingham Biomedical Research Centre, Nottingham) | ENG (National Institute for Health Research Nottingham Biomedical Research Centre, Nottingham) | USA (School of Clinical Psychology, American School of Professional Psychology at Argosy University, Alameda, California) |
| **Description** | Assessment of perceived benefit afforded by hearing aids. | Assessment of the perceived social impact of hearing loss. | Assessment of hearing-related social participation restrictions. | Assessment of the severity of subjective hyperacusis impact. |
| **Study population**  ***mean age, SD, age range, women (%)*** | 75 subjects (patients suffering sensorineural hearing loss using hearing aids): 71.7yrs, ±4yrs, range: 65-80yrs. | 116 subjects (hearing loss sufferers): 63.04yrs, ±12.09yrs, range: 21-94yrs, women: 47.4%. | Item reduction group 279 subjects (hearing loss sufferers): 65.67yrs, ±12.73yrs, range 24-89yrs, women: 44.1%. Validation group: 102 subjects (hearing loss sufferers): 60.92yrs, ±10.53yrs, range 25-90yrs, women: 47.1%. | 450 subjects (online recruited sample): 34.8 yrs, ±1.6yrs, women: 58%. |
| **Item development** | Items are all from Hearing Aid Performance Inventory. Item selection is based on response rate to individual items on the HAPI. | Items are derived from the Social Perceptions subscale of the Social Participation Restrictions Questionnaire. | Items are generated through literature review and individual, semi-structured interviews with adults with hearing loss, clinicians, and academics. | Items are generated based on literature review and patient interviews and health care professionals interviews. |
| **Items** | 38 items | 05 items | 19 items | 25 items |
| **Translations** | USA. | ENG. | ENG. | USA. |

|  | **Hearing in Real-Life Environments** | **Acceptance and Action Questionnaire-Adult Hearing Loss (AAQ-AHL)** | **University of Rhode Island Change Assessment - Hearing Loss** | **Hearing Satisfaction Scale (Hearing Aid/Surgery)** |
| --- | --- | --- | --- | --- |
| **Author (year)** | Heinrich A (2019)^47^ | Ong CW (2019)^48^ | Laplante-Lévesque A (2013)^49^ | Stewart MG (1997)^50^ |
| **Country (institution)** | ENG (Medical Research Council Institute of Hearing Research, School of Medicine, The University of Nottingham) | USA (Department of Psychology, Utah State University, Logan, Utah) | AUS (School of Health and Rehabilitation Sciences, University of Queensland) | USA (The Bobby R. Alford Department of Otorhinolaryngology and Communicative Sciences, Baylor College of Medicine, Houston, Texas) |
| **Description** | Assessment of self-reported speech perception, sound localization, and the socio-emotional consequences of hearing impairment in the context of social functioning. | Assessment of psychological inflexibility in hearing loss sufferers. | Assessment of stages of change in auditory rehabilitation in adults with acquired hearing impairment. | Assessment of satisfaction with hearing from an emotional/ social-situational standpoint in patients with conductive hearing loss. |
| **Study population**  ***mean age, SD, age range, women (%)*** | 631 subjects, 2 groups (community-dwelling older adults in Finland (FIN) and United Kingdom (UK)): 581 subjects (FIN): 82yrs, ±4.0yrs, range: 76-91yrs, women 63%. 50 subjects (UK): 69yrs, ±6.4yrs, range: 61-86yrs, women: 56%. | 264 subjects (hearing loss sufferers): 50.9yrs, ±17.6yrs. | 153 subjects (persons aged 50yrs and older seeking hearing help for the first time): 70.74 yrs, ±7.45yrs, women: 30.72%. | 47 subjects (patients recently treated for conductive hearing loss with either a hearing aid or surgery (stapedectomy)): 52,3yrs, 30-82 yrs, women: 61.7%. |
| **Item development** | Items are based on a collection of items from the APHAB, SSQ, and HHIE. | Items are based on the Tinnitus Acceptance Questionnaire (Westin,2008): items were adapted to change their focus from “chronic tinnitus” to “hearing loss.” | Items are derived from the pre contemplation, contemplation, and action stages from the University of Rhode Island Change Assessment questionnaire with replacing the words ‘my problem’ in each item by ‘my hearing problem’. | No description of item generation. |
| **Items** | 15 items | 12 items | 24 items | 11 items |
| **Translations** | ENG, FIN. | USA. | USA. | USA. |

|  | **Autophony Index (26-items)** | **Autophony Index (5-items)** | **Hearing Beliefs Questionnaire** | **Hearing Screening Inventory (HSI)** |
| --- | --- | --- | --- | --- |
| **Author (year)** | Crane BT (2010)^51^ | Crane BT (2010)^51^ | Saunders GH (2013)^52^ | Coren S (1992)^53^ |
| **Country (institution)** | USA (Johns Hopkins School of Medicine, Baltimore, Maryland) | USA (Johns Hopkins School of Medicine, Baltimore, Maryland) | USA (National Center for Rehabilitative Auditory Research, Portland VA Medical Center, Portland, Oregon) | CAN (Department of Psychology, University of British Columbia) |
| **Description** | Assessment of autophony severity. | Assessment of autophony severity. | Assessment of hearing health beliefs within the constructs of the health belief model. | Assessment of hearing loss. |
| **Study population**  ***mean age, SD, age range, women (%)*** | 19 subjects (patients with superior canal dehiscence syndrome undergoing superior canal dehiscence plugging): 48yrs, range: 29-66yrs, women: 47.3%. | 19 subjects (patients with superior canal dehiscence syndrome undergoing superior canal dehiscence plugging): 48yrs, range: 29-66yrs, women: 47.3%. | 223 subjects (random individuals (patients and accompanying persons) from a Medical Center waiting room): men 61.1yrs, ±14.1yrs, women: 57.5yrs, ±14yrs, women: 24.2%. | 422 subjects (general population):35,6yrs, range: 17-76yrs, women: 60.2% (cross-validation sample). |
| **Item development** | Items are based on the tinnitus reaction questionnaire. | Items are derived from 26-item Autophony Index. Item selection was done based on factor analysis. | Items are developed by the research team, adjusted after feedback from audiologists and laypersons. | Items generated based on frequently reported situations in which hearing loss impairs performance, from symptom lists, perceptual assays, previous hearing inventories, and case histories. |
| **Items** | 26 items | 05 items | 26 items | 12 items |
| **Translations** | USA. | USA. | USA. | CAN (English). |

|  | **Patulous Eustachian Tube Handicap Inventory 10** | **Noise Induced Hearing Loss Questionnaire** | **Hearing Ability Scale** | **Hearing Disability Questionnaire** |
| --- | --- | --- | --- | --- |
| **Author (year)** | Ikeda R (2017)^54^ | Rus RM (2008)^55^ | Schein JD (1970)^56^ | Lutman ME (1987)^57^ |
| **Country (institution)** | JPN (Department of Otolaryngology–Head and Neck Surgery, Tohoku University Graduate School of Medicine) | MYS (Department of Community Health and Family Medicine, Kulliyyah of Medicine, International Islamic University Malaysia) | USA (U.S. Department of Health Education and Welfare, Public Health Service, Health Services and Mental Health Administration) | ENG (MRC Institute of Hearing Research, Nottingham Clinical Outstation, General Hospital, Nottingham) |
| **Description** | Assessment of patulous Eustachian tube severity. | Assessment of knowledge, attitude and practice in relation to noise-induced hearing loss. | Assessment of hearing status. | Assessment of hearing disability and handicap. |
| **Study population**  ***mean age, SD, age range, women (%)*** | 89 subjects, 3 groups (patulous ET patients treated with silicone plug (SP), treated with self-instillation of physiological saline solution as conservative treatment group (CT) sensorineural hearing loss (SNHL) without findings of PET (SNHL)): 31 patients (SP): 48.6yrs, ±18.2 yrs, range: 21-75yrs, women: 51.61%. 29 patients (CT): 52.9yrs, ±22.3 yrs, range: 15-85yrs, women: 65.5%. 29 patients (SNHL): 64.7yrs, ±11.9 yrs, range: 47-85yrs, women: 65.5% | 83 subjects (sawmill workers): 48yrs, ±11.04yrs, women: 31.3%. | 1307 (adults attending an audiology clinic). | 1691 subjects (random sample of the UK adult population, including hearing aid wearers): range: 17-89yrs. |
| **Item development** | Items are generated by modifying the THI-12 items. | Items are generated based on literature review and a subject focus group meeting. | Items are generated by authors adjusted by patient feedback. | No description of item generation. |
| **Items** | 10 items | 42 items | 07 items | 09 items |
| **Translations** | ENG, JPN. | ENG, MYS. | USA | ENG. |

|  | **National Institute for Deafness and Communication Disorders Questionnaire** | **The Emotional Communication in Hearing Questionnaire (EMO-CHeQ)** | **Noise Exposure Questionnaire** | **1-Minute Noise Screen** |
| --- | --- | --- | --- | --- |
| **Author (year)** | McCullagh MC (2012)^58^ | Singh G (2019)^59^ | Johnson TA (2017)^60^ | Johnson TA (2017)^60^ |
| **Country (institution)** | USA (University of Michigan School of Nursing, Division of Health Promotion and Risk Reduction, Ann Arbor, Michigan) | CAN (Department of Psychology, Ryerson University, Toronto, Ontario) | USA (Hearing and Speech Department, University of Kansas Medical Center) | USA (Hearing and Speech Department, University of Kansas Medical Center) |
| **Description** | Assessment of a person’s need for medical examination of their hearing. | Assessment of experiences of hearing and handicap when listening to signals that contain vocal emotion information. | Assessment of an individual’s annual noise exposure. | Identification of individuals at highest risk for noise induced hearing loss. |
| **Study population**  ***mean age, SD, age range, women (%)*** | validatie: 103 subjects (farm operators): 53.48yrs, ±14.71 yrs, woman: 12%. | 586 participants (normal hearing adults and adults with hearing loss sufferers with and without hearing aids): women: 44.36%. | 114 subjects (college freshman): range: 18-19yrs, women: 57%. | 59 subjects (no description): range: 19-30yrs, women: 67,7%. |
| **Item development** | No description of item generation. | Items are derived from SAC, HHI-E, and SSQ, and patient discussion group, modified after researchers, clinicians, and patients feedback. | Items are based on the task-based questionnaire described by Neitzel, Q without name. (Neitzel, Contributions of Non-occupational Activities to Total Noise Exposure of Construction Workers). | No description of item generation. |
| **Items** | 10 items | 16 items | 11 items | 03 items |
| **Translations** | USA. | CAN (English). | USA. | USA. |

|  | **Nursing Home Hearing Handicap Index - Self-Version** | **Expected Consequences of Hearing Aid Ownership** | **Hearing Implant Sound Quality Index** | **Spatial Hearing Questionnaire** |
| --- | --- | --- | --- | --- |
| **Author (year)** | Show RL (1977)^61^ | Cox RM (2000)^62^ | Amann E (2014)^63^ | Tyler RS (2009)^64^ |
| **Country (institution)** | USA (Idahoh State University, Pocatello, Idaho) | USA (School of Audiology and Speech-Language Pathology, University of Memphis, and tDepartment of Veterans Affairs Medical Center, Memphis, Tennessee) | AUT (Clinical Research Department, MED-EL, Innsbruck) | USA (Department of Otolaryngology–Head and Neck Surgery, The University of Iowa, Iowa City, Iowa) |
| **Description** | Assessment of hearing handicap in geriatric nursing home residents. | Assessment of pre-fitting expectations on hearing aid ownership. | Assessment of auditory benefit in everyday listening situations in cochlear implant users. | Assessment of spatial hearing abilities. |
| **Study population**  ***mean age, SD, age range, women (%)*** | 105 subjects (nursing home residents): 80yrs, women: 79%. | 54 subjects (hearing aid owners): women: 25%. | 75 subjects (cochlear implant users): women: 49.3%. | 142 subjects (uni- and bilateral CI wearers): range: 18-89yrs, male subjects (54.2yrs, ±15.8yrs) and female (55.8yrs, ±15.4yrs), women: 54.2%. |
| **Item development** | Items are derived from a list of an unpublished study by Tannahil and Schow. | Each item for the ECHO was constructed by slightly rewording an item of the SADL. For the ECHO item, the SADL question was transformed into a statement of expectation. | Items are generated by an expert panel (audiologists, speech therapists, a psychologist and a bio-statician), partly from existing questionnaires, with help of patient input. | No description of item generation. |
| **Items** | 10 items | 15 items | 19 items | 24 items |
| **Translations** | USA, Persian. | USA, GER, DNK. | ENG, AUT, ESP, BRA, NLD. | USA, NLD, FRA, KOR, CHN (Mandarin), Persian. |

|  | **Hearing Participation Scale** | **Nijmegen Cochlear Implant Questionnaire** | **Cochlear Implant Function Index** | **Comprehensive Cochlear Implant Questionnaire** |
| --- | --- | --- | --- | --- |
| **Author (year)** | Hawthorne G (2002)^65^ | Hinderink JB (2000)^66^ | Coelho DH (2009)^67^ | King N (2014)^68^ |
| **Country (institution)** | AUS (Australian Centre for Posttraumatisch Mental Health, Department of Psychiatry, The University of Melbourne, Melbourne, Victoria) | NLD (Department of Otorhinolaryngology, University Hospital Nijmegen, St Radboud) | USA (Department of Otolaryngology, New York University School of Medicine, 550 First Avenue, New York) | USA (New York Eye and Ear Infirmary, Department of Otolaryngology, New York, New York) |
| **Description** | Assessment of self-esteem, social handicap, and hearing handicap after cochlear implantation.  *Abbreviated version of the Glasgow Hearing Status Inventory.* | Assessment health-related quality of life in cochlear implant users. | Assessment of cochlear implant auditory effectiveness in real world situations. | Assessment of changes in quality of life after receiving a second cochlear implant. |
| **Study population**  ***mean age, SD, age range, women (%)*** | 202 subjects (deafened adults with and without cochlear implants): women: 51.9%. | 91 subjects, 2 groups (CI-implantees (CI) and controls( C)): 45 subjects (CI): 50yrs, ±16yrs, women: 54%. 46 subjects (C): 51yrs, ±16yrs, women: 40%. | 245 patients (adult CI-users): 52.2yrs, range: 19-84yrs. | 54 subjects (adult English speaking cochlear implant wearers): 52yrs, ±17yrs, women: 57%. |
| **Item development** | Items are derived from Glasgow Health Status Inventory. | Items are derived from patient interviews, existing (published) questionnaires. | Items constructed by the authors (CI surgeons, CI auditory rehabilitationists and research psychologists), and patient feedback. | Items are developed based on literature review and interviews with the institution’s CI team (surgeons, audiologists, and speech pathologists). |
| **Items** | 11 items | 60 items | 22 items | 28 items |
| **Translations** | AUS. | ENG, NLD, BRA, GER, ITA, ESP, POL, FRA, CHN (not specified). | USA, NLD. | USA. |

|  | **Spatial Hearing Questionnaire - Short Form** | **Subjective Questionnaire for Early-Deafened CI users** | **The Music-Related QoL questionnaire** | **Attitude Questionnaire** |
| --- | --- | --- | --- | --- |
| **Author (year)** | Ou H (2017)^69^ | Debruyne J (2017)^70^ | Dritsakis G (2017)^71^ | Van de Brink RHS (1995)^72^ |
| **Country (institution)** | USA (Department of Communication Sciences and Disorders, Illinois State University, Normal) | NLD (Department of Otorhinolaryngology, Head and Neck Surgery, Maastricht University Medical Center, Maastricht) | ENG (Institute of Sound and Vibration Research, University of Southampton) | NLD (Rijksuniversiteit Groningen, Groningen) |
| **Description** | Assessment of spatial-hearing ability.  *Abbreviated version of spatial hearing questionnaire.* | Assessment of self-perceived benefit (primary sound processing, sense of safety, ease of communication, social aspects of hearing, and self-confidence.) in early-deafened CI users. | Assessment of music perception and music engagement, and their importance in cochlear implant users. | Assessment of attitude  toward the impairment and the possibility of using a hearing aid. |
| **Study population**  ***mean age, SD, age range, women (%)*** | 170 subjects, 3 groups (normal hearing (NH), mild to moderately severe sensorineural hearing loss (MSHL),uni- and bilateral cochlear imlantees (CI)): 51 subjects (NH): 34.2yrs, ± 14.2yrs, women: 86.3%. 47 subjects (MSHL): 57.8yrs, ±18.3yrs, women: 51.1%. 72 subjects (CI): 60.4yrs, ±14.2yrs, women 60.8%. | 27 subjects (unilateral CI implantation, acquisition of deafness or severe hearing impairment before the age of 5yrs and implantation in adulthood): 45yrs, range: 20–71yrs, women: 37.03%. | 147 subjects (adult CI-users, pre- and post lingually deafened): 56,69yrs, ±: 16.02yrs, rangen: 18-84yrs, women: 60.54%. | 198 subjects (hearing aid candidates) 73.9yrs, ±7.9yrs, range: 60-95yrs, women: 52%. |
| **Item development** | Items are derived from the Spatial Hearing Questionnaire. Item selection based on factor analysis. | Items are generated based on interviews with early deafened CI-users. | Items are generated on the basis of focus groups input with adult CI users adjusted by expert input (ENT-doctors, musical therapist, audiologists, researchers). | Items are generated by the authors based on the revised Health Belief Model. |
| **Items** | 06 items | 50 items | 36 items | 46 items |
| **Translations** | USA. | ENG, NLD. | ENG. | ENG, NLD |

|  | **Attitudes Towards Loss of Hearing Questionnaire Saunders** | **Hearing Attitudes in Rehabilitation Questionnaire** | **Self-efficacy for Situational Communication Management** | **Hearing Disability and Handicap Scale** |
| --- | --- | --- | --- | --- |
| **Author (year)** | Saunders GH (1996)^73^ | Hallam RS (1996)^74^ | Jennings MB (2005)^75^ | Hétu R (1994)^77^ |
| **Country (institution)** | USA (Lexington Center (G.H.S.), 75th Street & 30th Ave, Jackson Heights, Queens, New York) | ENG (Department of Psychology, University of East London, Romford Road, London) | CAN (National Centre for Audiology, School of Communication Sciences and Disorders and Graduate Program in Health and Rehabilitation Sciences, Faculty of Health Sciences, Western University, London, Ontario) | CAN (Groupe d'acoustique de I'Universite de Montreal) |
| **Description** | Assessment of attitudes toward hearing loss and hearing aids. | Assessment of the patient’s view of the effects of hearing impairment on the way that self is perceived, and the perception of others’ views of self as a hearing impaired person likely to have to wear a visible sign of that impairment, a hearing aid. | Assessment of perceived self-efficacy for managing communication in adults with acquired hearing loss. | Assessment of auditory disability and handicap in persons with noise-induced hearing loss.  *Abbreviated version of the Hearing Measurement Scale.* |
| **Study population**  ***mean age, SD, age range, women (%)*** | 226 subjects (uni and bilateral hearing impaired patients (sensorineural, conductive and mixed)): 70.5yrs, ±7.53, range: 37-87yrs, women: 0%. | 140 subjects (patients referred for potential hearing aid fitting): 74.2yrs, ±10.38, range: 36-96yrs, women: 55%. | Validation study^76^: 338 subjects (adult hearing loss sufferers both with and without hearing aids from 50yrs and up): 73.8yrs, range: 50-93yrs, women: 40.8%. | Validation study^78^:168 subject (NIHL-sufferers): 54yrs, ±11yrs, range: 17-73yrs, women: 0%. |
| **Item development** | Items are derived from Brooks’ ALHQ adjusted to American-English. | Items are derived from the ‘attitudes towards loss of hearing questionnaire’ van Brooks, with the addition of extra questions from authors/experts (in audiological rehabilitation). | Items are developed based on communication difficulties described in the literature, hearing-related self-report instruments, and the first author’s clinical experience.^76^ | Items are derived from the Hearing Measurement Scale. Some items were modified. |
| **Items** | 24 items | 40 items | 20 items | 20 items |
| **Translations** | USA. | ENG, NLD. | CAN. | ENG, FRA, SWE, NOR. |

|  | **Youth Attitude To Noise Scale** | **Five-Minute Hearing Test** | **Cochlear Implant Quality of Life -10 Profile (CIQOL-10 Global)** | **Cochlear Implant Quality of Life -35 Profile (CIQOL-35 Profile)** |
| --- | --- | --- | --- | --- |
| **Author (year)** | Olsen SE (2004)^79^ | American Academy of Otolaryngology-Head & Neck Surgery (1990)^81^ | McRackan TR(2019)^83^ | McRackan TR(2019)^83^ |
| **Country (institution)** | SWE (Department of Psychology, University of Goteborg, Sweden) | USA (American Academy of Otolaryngology—Head & Neck Surgery.) | USA (Department of Otolaryngology—Head and Neck Surgery, Medical University of South Carolina, Charleston) | USA (Department of Otolaryngology—Head and Neck Surgery, Medical University of South Carolina, Charleston) |
| **Description** | Assessment of adolescent’s attitudes towards noise. | Assessment of hearing loss among the elderly population. | Assessment of quality of life in adult cochlear implant users. | Assessment of quality of life in adult cochlear implant users. |
| **Study population**  ***mean age, SD, age range, women (%)*** | Validation study^80^: 245 subjects (high school students): 15.7yrs, range: 14-18yrs, women: 51%. | Validation study^82^: 558 subjects (regular members of the Thai population): 54.5yrs, range: 18-87yrs, women: 68%. | 371 subjects (CI-users): 59.5yrs, ±14.9yrs, range: 19-88yrs, women: 59.8%. | 371 subjects (CI-users): 59.5yrs, ±14.9yrs, range: 19-88yrs, women: 59.8%. |
| **Item development** | No description of item generation. | No description of item generation. | Items originate from a CI QoL item bank based on input from focus groups with CI-users the literature. | Items originate from a CI QoL item bank based on input from focus groups with CI-users the literature. |
| **Items** | 19 items | 15 items | 10 items | 35 items |
| **Translations** | ENG, SWE, BRA. | USA, ITA, THA. | USA. | USA. |

References:

1. Khalfa S, Dubal S, Veuillet E, Perez-Diaz F, Jouvent R, Collet L. Psychometric normalization of a hyperacusis questionnaire. ORL J Otorhinolaryngol Relat Spec. 2002;64(6):436-42.

2. Tortorella F, Pavaci S, Fioretti AB, Masedu F, Lauriello M, Eibenstein A. The short hyperacusis questionnaire: A tool for the identification and measurement of hyperacusis in the Italian tinnitus population. Audiol Res. 2017;7(2):182.

3. Dauman R, Bouscau-Faure F. Assessment and amelioration of hyperacusis in tinnitus patients. Acta Otolaryngol. 2005;125(5):503-9.

4. Cox RM, Alexander GC. The abbreviated profile of hearing aid benefit. Ear Hear. 1995;16(2):176-86.

5. Cox RM, Alexander GC. Measuring Satisfaction with Amplification in Daily Life: the SADL scale. Ear Hear. 1999;20(4):306-20.

6. Yueh B, McDowell JA, Collins M, Souza PE, Loovis CF, Deyo RA. Development and validation of the effectiveness of [corrected] auditory rehabilitation scale. Arch Otolaryngol Head Neck Surg. 2005;131(10):851-6.

7. Cox R, Hyde M, Gatehouse S, Noble W, Dillon H, Bentler R, et al. Optimal outcome measures, research priorities, and international cooperation. Ear Hear. 2000;21(4 Suppl):106s-15s.

8. Diges I, Simon F, Cobo P. Assessing Auditory Processing Deficits in Tinnitus and Hearing Impaired Patients with the Auditory Behavior Questionnaire. Front Neurosci. 2017;11:187.

9. Cox RM, Gilmore C. Development of the Profile of Hearing Aid Performance (PHAP). J Speech Hear Res. 1990;33(2):343-57.

10. Purdy SC, Jerram JC. Investigation of the profile of hearing aid performance in experienced hearing aid users. Ear Hear. 1998;19(6):473-80.

11. Cox RM, Gilmore C, Alexander GC. Comparison of two questionnaires for patient-assessed hearing aid benefit. J Am Acad Audiol. 1991;2(3):134-45.

12. Kim G, Na W, Kim G, Han W, Kim J. The development and standardization of Self-assessment for Hearing Screening of the Elderly. Clin Interv Aging. 2016;11:787-95.

13. Palmer CV, Mueller GH, Moriarty M. Profile of Aided Loudness: A validation procedure. Hearing J. 1999;52 (6):34,36,40-42

14. West RL, Smith SL. Development of a hearing aid self-efficacy questionnaire. Int J Audiol. 2007;46(12):759-71.

15. Smith SL, Pichora-Fuller KM, Watts KL, La More C. Development of the Listening Self-Efficacy Questionnaire (LSEQ). Int J Audiol. 2011;50(6):417-25.

16. Cox RM, Alexander GC, Xu J. Development of the Device-Oriented Subjective Outcome (DOSO) scale. J Am Acad Audiol. 2014;25(8):727-36.

17. Korkmaz MH, Bayir O, Er S, Isik E, Saylam G, Tatar EC, et al. Satisfaction and compliance of adult patients using hearing aid and evaluation of factors affecting them. Eur Arch Otorhinolaryngol. 2016;273(11):3723-32.

18. Demorest ME, Wark DJ, Erdman SA. Development of the screening test for hearing problems. Am J Audiol. 2011;20(2):100-10.

19. Espmark AK, Rosenhall U, Erlandsson S, Steen B. The two faces of presbyacusis: hearing impairment and psychosocial consequences. Int J Audiol. 2002;41(2):125-35.

20. Ventry IM, Weinstein BE. The hearing handicap inventory for the elderly: a new tool. Ear Hear. 1982;3(3):128-34.

21. Lichtenstein MJ, Bess FH, Logan SA. Validation of screening tools for identifying hearing-impaired elderly in primary care. Jama. .1988;259(19):2875-8.

22. Newman CW, Weinstein BE, Jacobson GP, Hug GA. The Hearing Handicap Inventory for Adults: psychometric adequacy and audiometric correlates. Ear Hear. 1990;11(6):430-3.

23. Newman CW, Weinstein BE, Jacobson GP, Hug GA. Test-retest reliability of the hearing handicap inventory for adults. Ear Hear. 1991;12(5):355-7.

24. Noble WG. The hearing measurement scale as a paper-pencil form: preliminary results. J Am Aud Soc. 1979;5(2):95-106.

25. Kramer SE, Kapteyn TS, Festen JM, Tobi H. Factors in subjective hearing disability. Audiology. 1995;34(6):311-20.

26. Meijer AG, Wit HP, TenVergert EM, Albers FW, Muller Kobold JE. Reliability and validity of the (modified) Amsterdam Inventory for Auditory Disability and Handicap. Int J Audiol. 2003;42(4):220-6.

27. High WS, Fairbanks G, Glorig A. Scale for Self-Assessment of Hearing Handicap. J Speech Hear Disord. 1964;29(3):215-30

28. Demorest ME, Erdman SA. Development of the communication profile for the hearing impaired. J Speech Hear Disord. 1987;52(2):129-43.

29. Gatehouse S, Noble W. The Speech, Spatial and Qualities of Hearing Scale (SSQ). Int J Audiol. 2004;43(2):85-99.

30. Noble W, Jensen NS, Naylor G, Bhullar N, Akeroyd MA. A short form of the Speech, Spatial and Qualities of Hearing scale suitable for clinical use: the SSQ12. Int J Audiol. 2013;52(6):409-12.

31. Show RL, Nerbonne MA. Hearing Handicap and Denver Scales; Application, Categories, Interpretation. J Acad of Rehabil Audiol. 1980;13: 66-77.

32. Tuley MR, Mulrow CD, Aguilar C, Velez R. A critical reevaluation of the Quantified Denver Scale of Communication Function. Ear Hear. 1990;11(1):56-61.

33. Giolas TG, Owens E, Lamb SH, Schubert ED. Hearing performance inventory. J Speech Hear Disord. 1979;44(2):169-95.

34. Ringdahl A, Eriksson-Mangold M, Andersson G. Psychometric evaluation of the Gothenburg Profile for measurement of experienced hearing disability and handicap: applications with new hearing aid candidates and experienced hearing aid users. Br J Audiol. 1998;32(6):375-85.

35. VK CM, Molander P, Ronnberg J, Andersson G, Lunner T. The acceptance of hearing disability among adults experiencing hearing difficulties: a cross-sectional study. BMJ Open. 2014;4(1):e004066.

36. Schow RL, Nerbonne MA. Communication screening profile: use with elderly clients. Ear Hear. 1982;3(3):135-47.

37. Bonetti L, Simunjak B, Franic J. Validation of self-reported hearing loss among adult Croatians: the performance of the Hearing Self-Assessment Questionnaire against audiometric evaluation. Int J Audiol. 2018;57(1):1-9.

38. Owens E, Raggio M. Performance inventory for profound and severe loss (PIPSL). J Speech Hear Disord. 1988;53(1):42-56.

39. Hidalgo JL, Gras CB, Lapeira JM, Martinez IP, Verdejo MA, Rabadan FE, et al. The Hearing-Dependent Daily Activities Scale to evaluate impact of hearing loss in older people. Ann Fam Med. 2008;6(5):441-7.

40. Kaplan H, Bally SJ, Brandt F. Communication Self-Assessment Scale Inventory for Deaf Adults. J Am Acad Audiol. 1991;2(3):164-82.

41. Lamb SH, Owens E, Schubert ED. The revised form of the Hearing Performance Inventory. Ear Hear. 1983;4(3):152-7.

42. Demeester K, Topsakal V, Hendrickx JJ, Fransen E, van Laer L, Van Camp G, et al. Hearing disability measured by the speech, spatial, and qualities of hearing scale in clinically normal-hearing and hearing-impaired middle-aged persons, and disability screening by means of a reduced SSQ (the SSQ5). Ear Hear. 2012;33(5):615-6.

43. Schum DJ. Responses of elderly hearing aid users on the hearing aid performance inventory. J Am Acad Audiol. 1992;3(5):308-14.

44. Heffernan E, Habib A, Ferguson M. Evaluation of the psychometric properties of the social isolation measure (SIM) in adults with hearing loss. Int J Audiol. 2019 Jan;58(1):45-52.

45. Heffernan E, Coulson NS, Ferguson MA. Development of the Social Participation Restrictions Questionnaire (SPaRQ) through consultation with adults with hearing loss, researchers, and clinicians: a content evaluation study. Int J Audiol. 2018;57(10):791-9.

46. Greenberg B, Carlos M. Psychometric Properties and Factor Structure of a New Scale to Measure Hyperacusis: Introducing the Inventory of Hyperacusis Symptoms. Ear Hear. 2018;39(5):1025-34.

47. Heinrich A, Mikkola TM, Polku H, Tormakangas T, Viljanen A. Hearing in Real-Life Environments (HERE): Structure and Reliability of a Questionnaire on Perceived Hearing for Older Adults. Ear Hear. 2019;40(2):368-80.

48. Ong CW, Whicker JJ, Muñoz K, Twohig MP. Measuring psychological inflexibility in adult and child hearing loss. Int J Audiol. 2019;58(10):643-650.

49. Laplante-Levesque A, Hickson L, Worrall L. Stages of change in adults with acquired hearing impairment seeking help for the first time: application of the transtheoretical model in audiologic rehabilitation. Ear Hear. 2013;34(4):447-57.

50. Stewart MG, Jenkins HA, Coker NJ, Jerger JF, Loiselle LH. Development of a new outcomes instrument for conductive hearing loss. Am J Otol. 1997;18(4):413-20.

51. Crane BT, Lin FR, Minor LB, Carey JP. Improvement in autophony symptoms after superior canal dehiscence repair. Otol Neurotol. 2010;31(1):140-6.

52. Saunders GH, Frederick MT, Silverman S, Papesh M. Application of the health belief model: development of the hearing beliefs questionnaire (HBQ) and its associations with hearing health behaviors. Int J Audiol. 2013;52(8):558-67.

53. Coren S, Hakstian AR. The development and cross-validation of a self-report inventory to assess pure-tone threshold hearing sensitivity. J Speech Hear Res. 1992;35(4):921-8.

54. Ikeda R, Kikuchi T, Oshima H, Miyazaki H, Hidaka H, Kawase T, et al. New Scoring System for Evaluating Patulous Eustachian Tube Patients. Otol Neurotol. 2017;38(5):708-13.

55. Rus RM, Daud A, Musa KI, Naing L. Knowledge, attitude and practice of sawmill workers towards noise-induced hearing loss in kota bharu, kelantan. Malays J Med Sci. 2008;15(4):28-34.

56. Schein JD, Gentile A, Haase KW. Development and evaluation of an expanded hearing loss scale questionnaire. Vital Health Stat 2. 1970(37):1-42.

57. Lutman ME, Brown EJ, Coles RR. Self-reported disability and handicap in the population in relation to pure-tone threshold, age, sex and type of hearing loss. Br J Audiol. 1987;21(1):45-58.

58. McCullagh MC. Validation of a questionnaire to identify hearing loss among farm operators. Noise Health. 2012;14(56):32-8.

59. Singh G, Liskovoi L, Launer S, Russo F. The Emotional Communication in Hearing Questionnaire (EMO-CHeQ): Development and Evaluation. Ear Hear. 2019;40(2):260-71.

60. Johnson TA, Cooper S, Stamper GC, Chertoff M. Noise Exposure Questionnaire: A Tool for Quantifying Annual Noise Exposure. J Am Acad Audiol. 2017;28(1):14-35.

61. Schow RL, Nerbonne MA. Assessment of hearing handicap by nursing home residents and staff. J Acad of Rehabil Audiol. 1977;10(2):2–12.

62. Cox RM, Alexander GC. Expectations about hearing aids and their relationship to fitting outcome. J Am Acad Audiol. 2000;11(7):368-82; quiz 407.

63. Amann E, Anderson I. Development and validation of a questionnaire for hearing implant users to self-assess their auditory abilities in everyday communication situations: the Hearing Implant Sound Quality Index (HISQUI19). Acta Otolaryngol. 2014;134(9):915-23.

64. Tyler RS, Perreau AE, Ji H. Validation of the Spatial Hearing Questionnaire. Ear Hear. 2009;30(4):466-74.

65. Hawthorne G, Hogan A. Measuring disability-specific patient benefit in cochlear implant programs: developing a short form of the Glasgow Health Status Inventory, the Hearing Participation Scale. Int J Audiol. 2002;41(8):535-44.

66. Hinderink JB, Krabbe PF, Van Den Broek P. Development and application of a health-related quality-of-life instrument for adults with cochlear implants: the Nijmegen cochlear implant questionnaire. Otolaryngol Head Neck Surg. 2000;123(6):756-65.

67. Coelho DH, Hammerschlag PE, Bat-Chava Y, Kohan D. Psychometric validity of the Cochlear Implant Function Index (CIFI): a quality of life assessment tool for adult cochlear implant users. Cochlear Implants Int. 2009;10(2):70-83.

68. King N, Nahm EA, Liberatos P, Shi Q, Kim AH. A new comprehensive cochlear implant questionnaire for measuring quality of life after sequential bilateral cochlear implantation. Otol Neurotol. 2014;35(3):407-13.

69. Ou H, Perreau A, Tyler RS. Development of a Shortened Version of the Spatial Hearing Questionnaire (SHQ-S) for Screening Spatial-Hearing Ability. Am J Audiol. 2017;26(3):293-300.

70. Debruyne J, Janssen M, Brokx J. Late Cochlear Implantation in Early-Deafened Adults: A Detailed Analysis of Auditory and Self-Perceived Benefits. Audiol Neurootol. 2017;22(6):364-76.

71. Dritsakis G, van Besouw RM, Kitterick P, Verschuur CA. A Music-Related Quality of Life Measure to Guide Music Rehabilitation for Adult Cochlear Implant Users. Am J Audiol. 2017;26(3):268-82.

72. Brink RHSVD. Attitude and illness behaviour in hearing impaired elderly. [S.l.]: [S.n.], 1995.

73. Saunders GH, Cienkowski KM. Refinement and psychometric evaluation of the Attitudes Toward Loss of Hearing Questionnaire. Ear Hear. 1996;17(6):505-19.

74. Hallam RS, Brooks DN. Development of the Hearing Attitudes in Rehabilitation Questionnaire (HARQ). Br J Audiol. 1996;30(3):199-213.

75. Jennings MB. (2005). Factors that influence outcomes from aural rehabilitation of older adults: The role of perceived self-efficacy. Unpublished doctoral dissertation. London, UK: University of Western Ontario.

76. Jennings MB, Cheesman MF, Laplante-Lévesque A. Psychometric properties of the self-efficacy for situational communication management questionnaire (SESMQ). Ear Hear. 2014;35(2):221-9.

77. Hétu R, Getty L, Philibert L, Noble WG, Stephens D. Development of a clinical tool for the measurement of severity of hearing disabilities and handicaps. J Speech Lang Pathol Audiol. 1994;18:83–95.

78. Barrenäs ML, Holgers KM. A clinical evaluation of the hearing disability and handicap scale in men with noise induced hearing loss. Noise Health. 2000;2(6):67-78.

79. Olsen, S. E. (2004). Psychological aspects of adolescents' perceptions and habits in noisy environments. Licentiate dissertation (unpublished). Department of Psychology, University of Goteborg, Sweden.

80. Zocoli AM, Morata TC, Marques JM. Youth Attitude to Noise Scale (YANS) questionnaire adaptation into Brazilian Portuguese. Braz J Otorhinolaryngol. 2009;75(4):485-92.

81. American Academy of Otolaryngology-Head & Neck Surgery. The five-minute hearing test. AAO-HNS Bulletin. 1990; 9(4):43.

82 Yimtae K, Kasemsiri P, Thanawirattananit P, Siripaopradith P. Validation of the Thai Five-Minute Hearing Test to Screen Hearing Loss in the Community. Audiol Neurootol. 2014;19(2):127-34.

83. McRackan TR, Hand BN, Velozo CA, Dubno JR. Cochlear Implant Quality of Life (CIQOL): Development of a Profile Instrument (CIQOL-35 Profile) and a Global Measure (CIQOL-10 Global). J Speech Lang Hear Res. 2019;62(9):3554-63.
