# Supplementary material for: Questionnaires in otology: a systematic mapping review
Source: Syst Rev. 2021 Apr 20;10:119. doi: 10.1186/s13643-021-01659-9 (PMC8059288; doi:10.1186/s13643-021-01659-9)
Supplement: Supplementary file 7 — Additional file 7. Multiple complaint questionnaires [file 13643_2021_1659_MOESM7_ESM.docx]

ADDITIONAL FILE 7 - MULTIPLE COMPLAINT QUESTIONNAIRES

|  | **Meniere’s Disease Patient Oriented Symptom-Severity Index** | **Chronic Otitis Media Questionnaire-12** | **Cambridge Otology Quality of Life Questionnaire** | **Penn Acoustic Neuroma Quality of Life Scale** |
| --- | --- | --- | --- | --- |
| **Author (year)** | Murphy MP (1999)^1^ | Phillips JS (2014)^2^ | Martin TP (2015)^3^ | Shaffer BT (2010)^4^ |
| **Country (institution)** | USA (Department of Otolaryngology-Head and Neck Surgery, University of Washington Medical Center, Seattle, Washington) | ENG (Norfolk & Norwich University Hospital, Norwich, Norfolk) | GBR (Worcestershire Royal Hospital, Worcester) | USA (Department of Otorhinolaryngology–Head and Neck Surgery, Hospital of the University of Pennsylvania, Philadelphia) |
| **Description** | Assessment of the impact of Meniere's disease on patients' lives. | Assessment of the quality of life in patients with chronic otitis media. | Assessment of the quality of life of patients attending otology clinics. | Assessment of quality of life in acoustic neuroma patients. |
| **Study population**  ***mean age, SD, age range, women (%)*** | 85 subjects (Meniere’s disease sufferers): 55.7yrs, range: 28-87yrs, women: 55-62%. | 50 subjects (adult patients with a history of active chronic otitis media): 50yrs, ±12yrs. | 203 subjects (adult patients attending otology clinics with: vertigo: 14%, active CSOM 14%, stable CSOM: 22%, otosclerosis: 7%, hearing loss only: 13%, tinnitus only: 8%, wax: 7%, otitis externa: 7%, Eustachian tube dysfunction: 2%, other: 6%). | 143 subjects, 2 groups (adult acoustic neuroma sufferers, not neurofibromatosis type 2 patients (AN) and control group of patients presenting at ENT/H&N department (C)): 143 subjects (AN): 59.1yrs, range: 20-88yrs, women: 51%.140 subjects (C): 51.4yrs, range: 18-84yrs, women: 43%. |
| **Item development** | Items generated based on professional experience with MD patients and consultation with colleagues. | All items were derived from the Chronic Ear Survey (CES), the Chronic Otitis Media Outcome Test 15 (COMOT-15) (5), and the Chronic OtitisMedia 5 (COM-5) questionnaires, revised by patient feedback and ultimate selection by expert panel. | Items were devised by the core research team and in part taken from existing validated questionnaires in the public domain. | Items constructed based on literature review, Hospital Anxiety and Depression Scale (HADS), interviews with expert caregivers. |
| **Items** | 20 items | 12 items | 24 items | 26 items |
| **Translations** | USA | ENG, SRB, PRT, NLD, RUS, Kannada | ENG | USA |

|  | **Eustachian Tube Dysfunction Questionnaire** | **Chronic Ear Survey** | **Otology Questionnaire Amsterdam** | **Screening Otologic Function Impairments** |
| --- | --- | --- | --- | --- |
| **Author (year)** | McCoul ED (2012)^5^ | Nadol JB (2000)^6^ | Bruinewoud EM (2018)^7^ | Weinstein BE (2013)^8^ |
| **Country (institution)** | USA (From the Department of Otolaryngology–Head and Neck Surgery and Department of Public Health, Weill Cornell Medical College, New York, New York) | USA (Department of Otology and Laryngology, Harvard Medical School, and Massachusetts Eye and Ear Infirmary, Boston, Massachusetts) | NLD (Otolaryngology- Head and neck Surgery, Section Ear & Hearing, VU University Medical Center) | USA (The Graduate Center, CUNY, New York, New York) |
| **Description** | Assessment of Eustachian tube disease-related symptoms. | Assessment of disease specific quality of life in patients with chronic suppurative otitis media. | Assessment of the severity of ear complaints and their impact on patients lives. | Identification of older adults requiring audiologic intervention. |
| **Study population**  ***mean age, SD, age range, women (%)*** | 75 subjects, 2 groups (adult patients diagnosed with ETD and a control group (C)): 50 subjects (ETD) 49.8yrs, ±13.9yrs, women: 52%. 25 subjects (C): 53.4yrs, ±11.2yrs, women: 48%. | 91 subjects (chronic active otitis media with cholesteatoma, 40.8%; chronic active otitis media without cholesteatoma, 23.8%; chronic inactive otitis media with frequent reactivation, 12.9%; and chronic inactive otitis media, 25.5%.): 44.3yrs, ±16yrs, women: approximately 50%. | 352 subjects (patients from the age of 16 and older visiting an ENT surgeon with an ear complaint): 49yrs, range: 16-93yrs, women: 50.3%. | 29 subjects (patients visiting an otolaryngology clinic): 72yrs, ±9.2yrs. |
| **Item development** | Literature review (Otitis Media 6-Item Quality-of-Life Survey (OM-6), the Nasal Obstruction Symptom Evaluation, and the 20-Item Sino-Nasal Outcomes Test (SNOT-20)) and item generation by the authors, adjusted according to patient feedback. | Items were generated by an expert panel of otologists. | Items were constructed based on patient interviews, revised by input from an expert panel (incl specialists in ear and hearing) or adapted from the Amsterdam Inventory for Auditory Disability and Handicap. | Items were drawn primarily from the THI, DHI, and HHI-E. |
| **Items** | 07 items | 13 items | 34 items | 10 items |
| **Translations** | USA, GER, CHN (Mandarin/Cantonese?), Hebrew. | USA, ITA, KOR, Chinese (Mandarin). | ENG, NLD. | USA. |

|  | **Menière’s Disease Outcome Questionnaire - Retrospective Version** | **Dizziness, tinnitus and taste disturbances questionnaire** | **Chronic Otitis Media Outcome Test 15** | **Gopen–Yang Superior Semicircular Canal Dehiscence Questionnaire** |
| --- | --- | --- | --- | --- |
| **Author (year)** | Kato BM (2004)^9^ | Mikkelsen KS (2017)^10^ | Baumann I (2009)^11^ | Voth BL (2018)^12^ |
| **Country (institution)** | USA (Kaiser Permanente Medical Center, Union City, California) | DNK (Department of Otorhinolaryngology, Head and Neck Surgery and Audiology, Rigshospitalet, Copenhagen) | GER (Universitäts-HNO-Klinik, Heidelberg) | USA (Department of Neurosurgery, David Geffen School of Medicine, University of California, Los Angeles) |
| **Description** | Assessment of quality of life in patients with Ménière’s disease (after surgery). | Assessment of dizziness,  tinnitus and taste disturbances in cochlear implantees. | Assessment of quality of life in individuals with chronic suppurative otitis media. | Assessment of subjective symptoms in patients undergoing surgical repair of superior semicircular canal dehiscence. |
| **Study population**  ***mean age, SD, age range, women (%)*** | 159 subjects (patients with Ménière’s disease who underwent endolymphatic sac decompression after failing a course of medical management (with diuretics, diet modification, and oral steroids when appropriate)): 52.4yrs, women: 53.5%, range 20-83yrs. | 77 subjects (cochlear implantees): women: 66.23%. | 121 subjects (patients with chronic suppurative mesotympanic or epitympanic otitis media): 48,8yrs, ±14.5yrs, range: 18-75yrs, women: 52.06%. | 23 subjects (patients undergoing surgical repair of superior semicircular canal dehiscence): 52.5yrs, ±12.7yrs, women: 52,2%. |
| **Item development** | Items were generated by authors based on clinical experience and previous outcomes work on Menieres Disease. | No description of item generation. | Items were generated by a group of experts. | Items are derived from the Rand 36-item Short-Form Health Survey (‘ SF-36’ ), Tinnitus Handicap Inventory and the Dizziness Handicap Inventory. |
| **Items** | 40 items | 15 items | 15 items | 68 items |
| **Translations** | USA. | ENG, DNK. | ENG, GER, Kannada. | USA. |

|  | **Dizziness Symptom Profile** | **Dokuz Eylül University Meniere’s Disease Disability Scale** | **Consumer Ear Disease Risk Assessment** |  |
| --- | --- | --- | --- | --- |
| **Author (year)** | Jacobson GP (2019)^13^ | Mutlu B (2018)^14^ | Kleindienst SJ (2017)^15^ |  |
| **Country (institution)** | USA (Department of Hearing and Speech Sciences, Division of Vestibular Sciences, Vanderbilt University School of Medicine, Nashville, Tennessee) | TUR (Department of Otorhinolaryngology, Unit of Hearing, Speech and Balance, Dokuz Eylül Univeristy School of Medicine, İzmir) | USA (Division of Audiology, Mayo Clinic Arizona, Scottsdale) |  |
| **Description** | Generation of a differential diagnosis in dizziness patients. | Assessment of quality of life in Meniere’s disease sufferers. | Assessment of ones risk for ear diseases associated with hearing loss. |  |
| **Study population**  ***mean age, SD, age range, women (%)*** | 162 subjects (patients evaluated for dizziness, vertigo, and unsteadiness): 54.86yrs, ±15.32yrs, range: 18-76 yrs, women: 58.64%. | 93 subjects (MD patients): 48.9yrs, ±12.1yrs, women: 51.6%. | 307 subjects (patients with ear or hearing-related complaints requiring neurotologic examination): 62.9yrs, ±9.8 years, women: 48%. |  |
| **Item development** | Items generated based on diagnostic criteria and consensus statement in literature and expert opinion. | Items generated based on disease characteristics, MD specific and neuro-otological questionnaires. | Items generated by neurology content experts refined through cognitive patient interviews. |  |
| **Items** | 31 items | 32 items | 15 items |  |
| **Translations** | USA. | ENG, TUR. | USA. |  |

References:

1. Murphy MP, Gates GA. Measuring the effects of Meniere's disease: results of the Patient-Oriented Severity Index (MD POSI) version 1. Ann Otol

Rhinol Laryngol. 1999;108(4):331-7.

1. Phillips JS, Haggard M, Yung M. A new health-related quality of life measure for active chronic otitis media (COMQ-12): development and initial

validation. Otol Neurotol. 2014;35(3):454-8.

1. Martin TP, Moualed D, Paul A, Ronan N, Tysome JR, Donnelly NP, et al. The Cambridge Otology Quality of Life Questionnaire: an otology-

specific patient-recorded outcome measure. A paper describing the instrument design and a report of preliminary reliability and validity. Clin

Otolaryngol. 2015;40(2):130-9.

1. Shaffer BT, Cohen MS, Bigelow DC, Ruckenstein MJ. Validation of a disease-specific quality-of-life instrument for acoustic neuroma: the Penn

Acoustic Neuroma Quality-of-Life Scale. Laryngoscope. 2010;120(8):1646-54.

1. McCoul ED, Anand VK, Christos PJ. Validating the clinical assessment of eustachian tube dysfunction: The Eustachian Tube Dysfunction

Questionnaire (ETDQ-7). Laryngoscope. 2012;122(5):1137-41.

6. Nadol JB, Jr., Staecker H, Gliklich RE. Outcomes assessment for chronic otitis media: the Chronic Ear Survey. Laryngoscope. 2000;110(3 Pt

3):32-5.

7. Bruinewoud EM, Kraak JT, van Leeuwen LM, Kramer SE, Merkus P. The Otology Questionnaire Amsterdam: a generic patient reported outcome

measure about the severity and impact of ear complaints. A cross-sectional study on the development of this questionnaire. Clin Otolaryngol.

2018;43(1):240-8.

8. Weinstein BE. Tool kit for screening otologic function of older adults1. Am J Audiol. 2013;22(1):179-82.

9. Kato BM, LaRouere MJ, Bojrab DI, Michaelides EM. Evaluating quality of life after endolymphatic sac surgery: The Meniere's Disease Outcomes

Questionnaire. Otol Neurotol. 2004;25(3):339-44.

10. Mikkelsen KS, Ovesen T, Swan CZ. Pre- and post-operative dizziness, tinnitus, and taste disturbances among cochlear implant recipients. J

Laryngol Otol. 2017;131(4):309-15.

11. Baumann I, Kurpiers B, Plinkert PK, Praetorius M. [Development and validation of the Chronic Otitis Media Outcome Test 15 (COMOT-15).

Measurement of health-related quality of life in patients with chronic otitis media]. Hno. 2009;57(9):889-95.

12. Voth BL, Sheppard JP, Barnette NE, Ong V, Nguyen T, Jacky Chen CH, et al. The Gopen-Yang Superior Semicircular Canal Dehiscence

Questionnaire: development and validation of a clinical questionnaire to assess subjective symptoms in patients undergoing surgical repair of

superior semicircular canal dehiscence. J Laryngol Otol. 2018;132(12):1110-8.

13. Jacobson GP, Piker EG, Hatton K, Watford KE, Trone T, McCaslin DL, et al. Development and Preliminary Findings of the Dizziness Symptom

Profile. Ear Hear. 2019;40(3):568-76.

14. Mutlu B, Kirkim G, Mungan Durankaya S, Gurkan S, Basokcu TO, Guneri EA. The Reliability and Validity of "Dokuz Eylul University Meniere's

Disease Disability Scale". J Int Adv Otol. 2018;14(2):304-11.

15. Kleindienst SJ, Zapala DA, Nielsen DW, Griffith JW, Rishiq D, Lundy L, et al. Development and Initial Validation of a Consumer Questionnaire to

Predict the Presence of Ear Disease. JAMA Otolaryngol Head Neck Surg. 2017;143(10):983-9.
